# Body Composition Phenotypes and Their Metabolic Associations in Treatment‐Naïve Patients With Newly Diagnosed Type 2 Diabetes: A DXA‐Based Cluster Analysis

**DOI:** 10.1002/edm2.70366

**Published:** 2026-10-19

**Authors:** Xintong Zhang, Rui Zhang, Tao Jiang

**Affiliations:** ^1^ Beijing Shijitan Hospital Capital Medical University Beijing China; ^2^ Department of Endocrinology, Beijing Shijitan Hospital Capital Medical University Beijing China

**Keywords:** body composition analysis, body composition phenotype, diabetes, diabetes heterogeneity, dual‐energy X‐ray absorptiometry, low muscle mass, obesity

## Abstract

**Background and Aims:**

We characterized body‐composition heterogeneity in treatment‐naive type 2 diabetes (T2DM) using dual‐energy X‐ray absorptiometry (DXA), and assessed whether cluster–metabolic associations persisted after accounting for sex‐related body‐composition differences.

**Methods:**

K‐means clustering of 50 DXA‐derived body‐composition variables classified 185 treatment‐naive T2DM patients. Variance decomposition, sex‐stratified analyses, sex‐stratified clustering, sex‐specific standardization clustering and multiple imputation (20 datasets) addressed sex confounding and missing data.

**Results:**

Four exploratory body‐composition clusters were identified: Low Fat–Low Muscle, Male‐Predominant Central Obesity (98.6% male), Female‐Predominant Low‐Muscle High‐Adiposity (98.0% female) and High Fat–High Muscle. Two clusters showed strong sex predominance; sex‐specific normalization reduced sex‐cluster dependence while preserving metabolic associations. In multiple‐imputation‐adjusted models, Clusters 2 and 4 had markedly higher MASLD odds (OR = 9.69 and 8.55, respectively), while Cluster 4 had lower osteoporosis (OR = 0.16) and higher hyperuricemia (OR = 6.36) odds versus Cluster 1. Bone turnover markers, diabetic retinopathy and diabetic kidney disease showed no significant associations.

**Conclusions:**

DXA‐based body‐composition clusters are associated with distinct metabolic and comorbidity profiles—particularly MASLD, osteoporosis and hyperuricemia—that persisted after sex‐specific standardization and multiple imputation. Given the sex–cluster overlap and modest silhouette width, these findings are hypothesis‐generating and require prospective validation.

## Graphical abstract

Using dual‐energy X‐ray absorptiometry clustering of 185 treatment‐naïve type 2 diabetes patients, we identified four body‐composition phenotypes. Central‐obesity and high‐fat–high‐muscle clusters carried the highest fatty liver disease and hyperuricemia risk, whereas the low‐muscle cluster carried the highest osteoporosis risk. These associations persisted after sex‐specific normalization, although clusters substantially overlapped with sex.

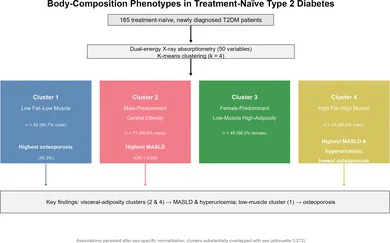

## 1 Introduction

Type 2 diabetes (T2DM) exhibits profound clinical heterogeneity, rendering conventional classification schemes insufficient for fully capturing biological heterogeneity [1]. Ahlqvist et al. identified five subtypes (SAID, SIDD, SIRD, MOD and MARD) based on clinical variables including age at onset, BMI, HbA1c, GADA, HOMA2‐B and HOMA2‐IR, with differential complication risks across subtypes [2]. Dennis et al. subsequently replicated these findings in independent cohorts [3], and Chinese cluster analyses have revealed similar metabolic heterogeneity [4].

Adipose tissue distribution and muscle mass modulate insulin sensitivity and metabolic risk through adipokine and myokine secretion [5, 6, 7, 8, 9]. Metabolic health depends not only on tissue mass but on anatomical distribution [10, 11]: gluteofemoral fat may confer protection, whereas visceral adiposity associates with adverse profiles. DXA‐derived body composition—which simultaneously quantifies regional fat mass, lean mass and bone mineral density—may capture dimensions complementary to clinical‐variable clustering.

This study employs unsupervised clustering to characterize body‐composition heterogeneity in treatment‐naive T2DM, profiling glucose homeostasis, β‐cell function and bone metabolism. Given the well‐established sexual dimorphism in body composition, we conducted variance decomposition and sex‐stratified analyses to evaluate whether cluster–metabolic associations persisted after accounting for sex‐related differences.

## 2 Methods

### 2.1 Research Population

Newly diagnosed, treatment‐naive T2DM patients were consecutively recruited from Beijing Shijitan Hospital (January 2020–June 2024). The study was approved by the Institutional Ethics Committee (sjtkyll‐lx‐2021(27)), registered at ChiCTR (ChiCTR1900022768) and conducted in accordance with the Declaration of Helsinki. All participants provided written informed consent.

### 2.2 Inclusion and Exclusion Criteria

Eligible participants were aged 18–80 years with treatment‐naive T2DM. Exclusion criteria were: (1) Type 1, gestational, or secondary diabetes (identified by autoantibody [GADA], history and C‐peptide); (2) conditions independently perturbing body composition and bone metabolism—active malignancy, end‐stage organ failure (NYHA Class IV, decompensated cirrhosis or CKD Stage 4–5) and malnutrition (weight loss > 10% over 6 months or BMI < 18.5 kg/m^2^); and (3) prior glucose‐lowering medication. These exclusions minimized confounding by catabolic or volume‐overload states.

### 2.3 Study Design and Data Collection

This cross‐sectional study recorded demographics, blood pressure and biochemical profiles. Glycemic indices (fasting and postprandial glucose, insulin, C‐peptide) were measured to calculate the AUC; HbA1c, HOMA‐IR and HOMA‐β were determined. Bone markers (25(OH)D, PTH, N‐MID, β‐CTX, P1NP) and total/regional fat mass, lean mass and BMD were quantified by DXA.

Plasma glucose was measured by the glucose oxidase method (AU5800, Beckman Coulter); insulin and C‐peptide by chemiluminescent immunoassay (ADVIA Centaur XP, Siemens); HbA1c by high‐performance liquid chromatography (Variant II Turbo, Bio‐Rad). HOMA‐IR = fasting insulin (μIU/mL) × fasting glucose (mmol/L)/22.5; HOMA‐β = 20 × fasting insulin (μIU/mL)/(fasting glucose [mmol/L] − 3.5). Areas under the curve were computed by the trapezoidal rule. Bone turnover markers (N‐MID, P1NP, β‐CTX) and PTH were assayed by electrochemiluminescence immunoassay (Cobas e601; Roche); 25(OH)D by chemiluminescent immunoassay.

### 2.4 Statistical Analysis

#### 2.4.1 Clustering Analysis Protocol

Fifty DXA‐derived variables (LArmFatMassg to AppenLeanBMCHt2kgm2) were selected a priori, encompassing regional fat mass, lean mass, total mass and fat percentage for the arms, legs, trunk, subtotal, head, total body, limbs, android and gynoid regions; VAT mass, volume and area; total body fat percentage; FMI; android‐to‐gynoid ratio; and trunk‐to‐limb fat mass ratio. All variables were *Z*‐score standardized. Given the exploratory nature of this study, all clinically relevant variables—including highly correlated ones representing meaningful regional distributions—were retained to capture multidimensional patterns. Principal component reduction was not applied to preserve clinically interpretable regional body‐composition features rather than maximize statistical separation. Robustness to feature redundancy was assessed by re‐clustering with 30 less‐correlated variables (*r* < 0.95), quantified by ARI.

Observations with missing body composition data were excluded, and outliers (> 3 SD) were removed, yielding 185 participants. Internal validation favoured K‐means over hierarchical clustering—Silhouette Width 0.272 vs. 0.222 and Calinski‐Harabasz index 79.05 vs. 67.25—though the modest silhouette value indicates limited discrete separation. The optimal cluster number (*k* = 4) was determined by the Silhouette and Elbow methods (nstart = 50). Bootstrap resampling (100 iterations) yielded Jaccard indices > 0.85 for all clusters (Figure 1). Given the low silhouette width, the four‐cluster solution is regarded as a pragmatic partition facilitating clinical description rather than definitive evidence of discrete categories.

**FIGURE 1:**
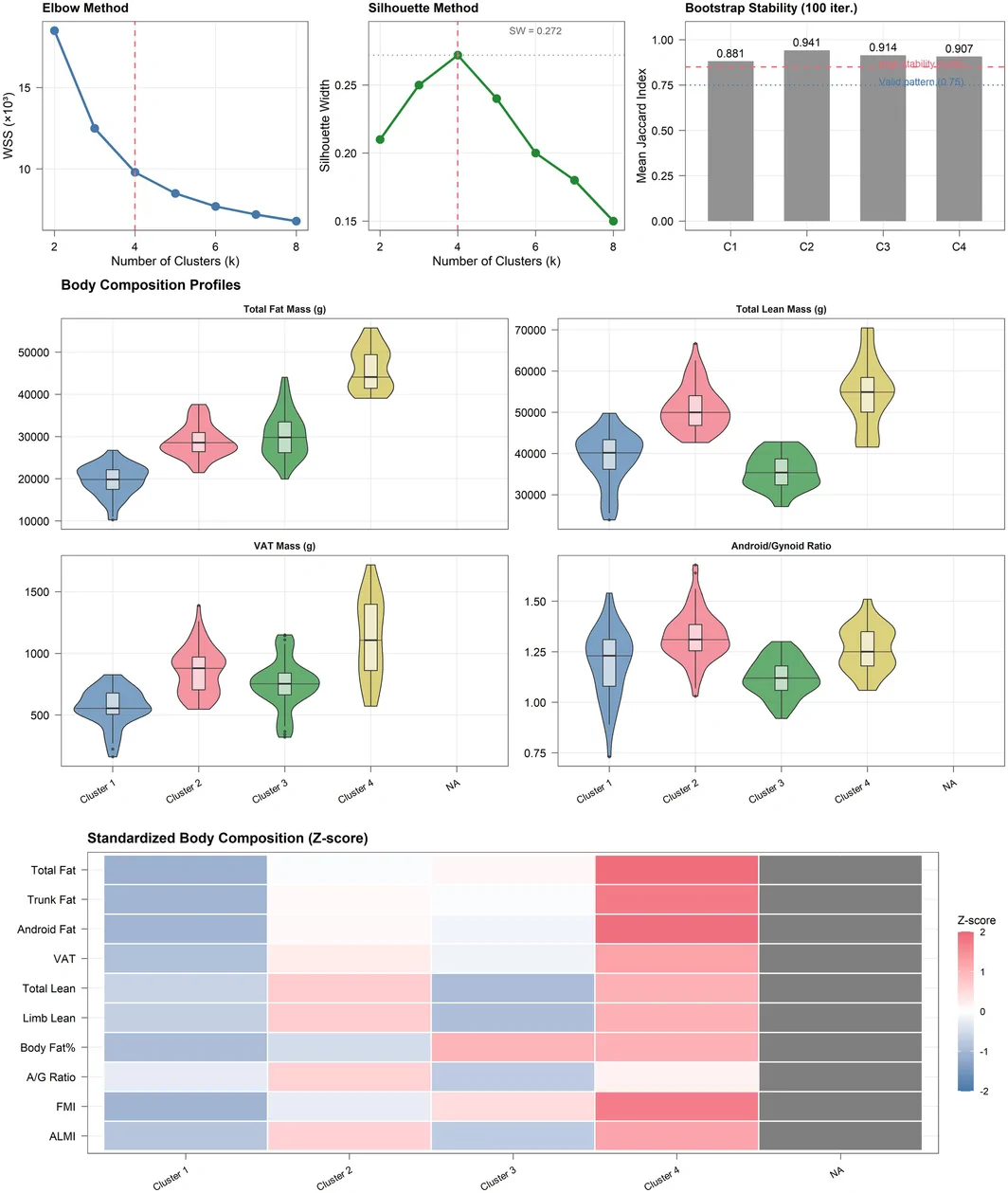
Clustering analysis and phenotypic characterization of treatment‐naive T2DM patients based on DXA‐derived body composition metrics. (A) Determination of the optimal number of clusters. Elbow Method (left): Within‐cluster sum of squares (WSS) against *k*, with inflection at *k* = 4. Silhouette Method (right): Average silhouette width peaks at *k* = 4 (SW = 0.272); the modest absolute value indicates limited discrete separation. (B) Key body composition variables across the four clusters. Violin plots with overlaid boxplots (median, IQR, 1.5×IQR whiskers) display the distribution of total fat mass, total lean mass, estimated VAT mass and the A/G ratio. These variables are presented descriptively without inferential statistics. (C) Heatmap of *Z*‐score standardized body composition profiles. Blue: Below cohort mean; red: Above cohort mean. Cluster 1 is characterized by globally low tissue masses; Cluster 2 by elevated central adiposity; Cluster 3 by high fat percentage with relatively low lean mass; Cluster 4 by universally elevated fat and lean mass. (D) Bootstrap validation of cluster stability (100 iterations). The red dashed line indicates Jaccard index > 0.85 (high bootstrap assignment stability); the blue dotted line indicates Jaccard index > 0.75 (valid pattern). All four clusters exceeded 0.85. A/G ratio, android‐to‐gynoid ratio; DXA, dual‐energy X‐ray absorptiometry; IQR, interquartile range; VAT, visceral adipose tissue.

#### 2.4.2 Statistical Inference

Analyses used SPSS 27.0 and R 4.4.3; all tests were two‐sided. Continuous variables were assessed for normality (Shapiro–Wilk) and presented as mean ± SD or median (Q1, Q3); categorical variables as *n* (%). Group comparisons used one‐way ANOVA or Kruskal–Wallis, with Bonferroni correction within each analysis set (*p*_adj = min[*p*_raw × *N*, 1]); Dunn's test was used for post hoc comparisons.

Multivariable linear regression assessed independent cluster–metabolic associations, adjusting for sex, age, smoking and alcohol consumption. For complication outcomes, variables significant in univariate analyses entered logistic regression models with cluster as the primary predictor, adjusting for age, sex, smoking and alcohol consumption.

#### 2.4.3 Sex‐Stratified Sensitivity Analyses

Given sexual dimorphism in body composition, we (1) cross‐tabulated sex by cluster, (2) repeated Kruskal–Wallis tests within sex strata (*n* ≥ 5 per cluster), (3) tested sex‐by‐cluster interactions and (4) compared cluster‐only versus cluster‐plus‐sex models to quantify sex's contribution to cluster‐related variance. To determine whether within‐sex body‐composition phenotypes exist, we additionally performed sex‐stratified *k*‐means clustering using the same 50 DXA variables, standardized within each sex (males, *k* = 3; females, *k* = 2), with the number of clusters chosen by the silhouette and elbow methods (Table S11).

#### 2.4.4 Variance Decomposition Analysis

To quantify the extent to which clusters recapitulate sex versus carry independent information, we performed variance decomposition for each of the 50 DXA‐derived variables, fitting three nested models—variable ~ cluster, variable ~ sex and variable ~ cluster + sex—and computing Δ*R* ^2^_cluster = *R* ^2^(cluster + sex) − *R* ^2^(sex) and Δ*R* ^2^_sex = *R* ^2^(cluster + sex) − *R* ^2^(cluster) (Table S4). Because cluster assignment was derived from DXA variables, variance decomposition was interpreted as quantifying the relative contribution of cluster labels versus sex rather than independent validation of cluster existence.

#### 2.4.5 Sex‐Specific Standardization Sensitivity Analysis

To assess whether the cluster structure was driven by sex‐related magnitude differences, we performed a sex‐specific standardization sensitivity analysis. Each DXA variable was *Z*‐score standardized separately within males and females, and *k*‐means clustering (*k* = 4, nstart = 50) was repeated on the pooled sex‐standardized data. Agreement between the original and sex‐standardized clusterings, and the association of each clustering with sex, were quantified by the Adjusted Rand Index (ARI). Cluster–outcome associations were re‐examined under sex‐specific standardization (Table S9, Figure S1).

#### 2.4.6 Missing Data Handling and Multiple Imputation

Substantial missing data were present for several complication and covariate variables (Tables S6 and S8): diabetic retinopathy (DR) 43.5%, carotid plaque 41.4%, limb arteriosclerosis 31.7% and MASLD 15.6%; smoking and drinking were missing in 11.8% and 11.3%. Complete‐case logistic regression models would retain only 96 (DR) to 156 (osteoporosis) of 186 participants, raising concerns about selection bias and reduced power.

We therefore employed multiple imputation by chained equations (MICE; R ‘mice’ 3.16.0), generating 20 imputed datasets with 20 iterations; 20 imputations were chosen based on the proportion of missing information and recommended practice for moderate missingness. Binary variables were imputed by logistic regression, continuous variables by predictive mean matching and cluster by polytomous regression. Models were fitted to each imputed dataset and pooled by Rubin's rules, with parallel complete‐case sensitivity analyses. Non‐binary complication coding was standardized to binary (DKD Stages 2/3/5, DR Grades 1/2, LEAD Grade 2 → 1).

## 3 Results

### 3.1 Baseline Characteristics

Baseline characteristics were compared across the four clusters. Sex distribution differed markedly (raw *p* < 0.001; adjusted *p* < 0.001): Cluster 2 was 98.6% male (70/71) and Cluster 3 was 98.0% female (48/49), whereas Clusters 1 and 4 were male‐skewed (66.7% and 65.2% male) (Figure 2A). Age was lower in Clusters 2 and 4 (raw *p* < 0.001; adjusted *p* = 0.005). Height, weight, BMI and waist circumference differed across clusters (all adjusted *p* < 0.001). Smoking and alcohol consumption also varied, with Cluster 3 showing the lowest prevalence of both; systolic and diastolic blood pressure did not differ (adjusted *p* = 1.000).

**FIGURE 2:**
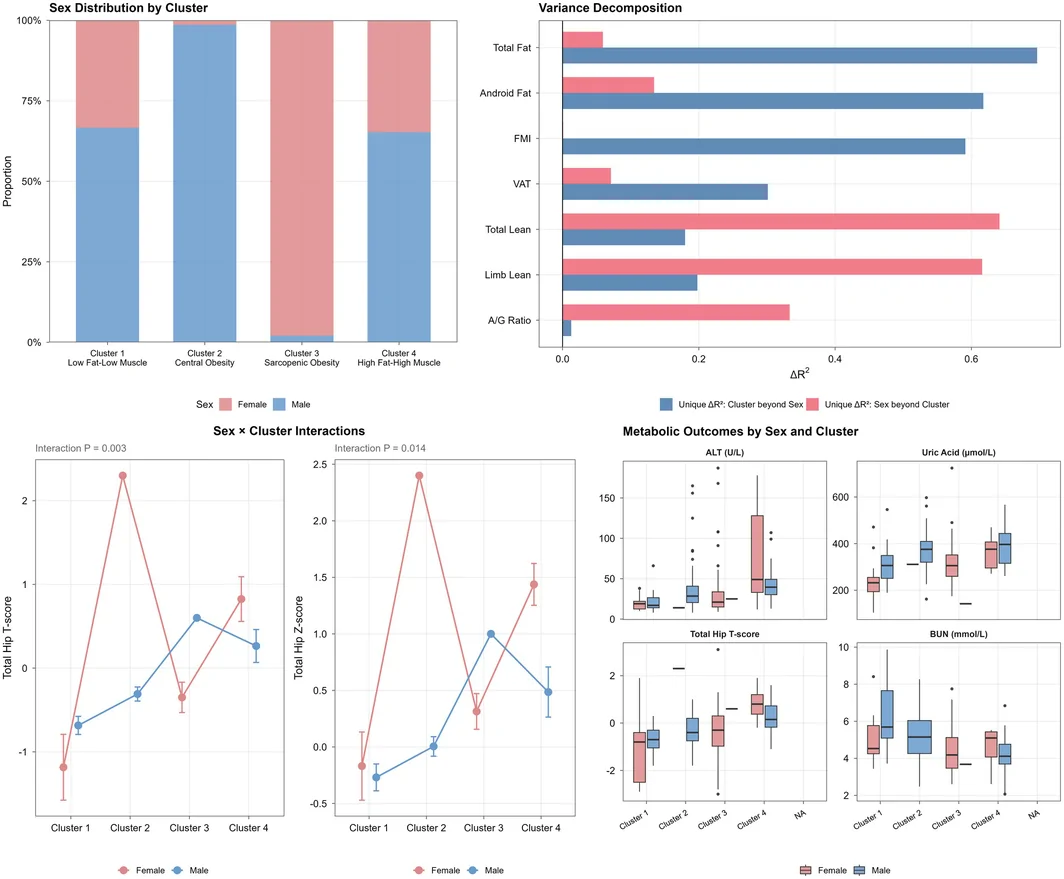
Sex‐cluster relationship and variance decomposition of DXA‐derived body composition variables. (A) Sex distribution across clusters. Stacked bars show proportions of females (pink) and males (blue); numbers indicate absolute counts. Clusters 2 and 3 exhibit near‐complete sex segregation (98.6% male, 98.0% female). (B) Variance decomposition of seven representative DXA variables. Blue bars: Unique variance contributed by cluster beyond sex (Delta‐R2_cluster). Red bars: Unique variance contributed by sex beyond cluster (Delta‐R2_sex). Values for fat distribution variables (median Delta‐R2_cluster = 0.59) indicate substantial cluster‐specific information beyond sex. (C) Sex × cluster interaction plots for total hip *T*‐score (*p* = 0.003) and total hip *Z*‐score (*p* = 0.014). Points represent mean ± SEM. (D) Sex‐stratified metabolic outcomes. Boxplots display ALT, uric acid, total hip *T*‐score and BUN by cluster and sex. Within‐sex comparisons with Bonferroni correction confirmed cluster‐associated differences persist within each sex stratum (see Section 3.2).

### 3.2 Sex‐Stratified Sensitivity Analyses and Variance Decomposition

Variance decomposition quantified the relative contribution of cluster labels and sex to DXA‐derived variability. Cluster labels accounted for substantially more variance than sex alone for most DXA variables, reflecting differences in multivariate body‐composition profiles rather than demonstrating independence from sex: for total fat mass, cluster explained 74.4% of variance beyond sex (*R* ^2^_cluster = 0.746, *R* ^2^_sex = 0.002) and for android fat mass Δ*R* ^2^_cluster = 0.692. For lean mass, contributions were more balanced (total lean Δ*R* ^2^_cluster = 0.251 vs. Δ*R* ^2^_sex = 0.104; A/G ratio 0.047 vs. 0.068) (Figure 2B, Table S4). Re‐clustering with 30 less‐correlated variables reproduced the original solution (ARI = 0.912), indicating robustness to feature redundancy.

Multivariable linear regression comparing cluster‐only versus cluster‐plus‐sex models confirmed that sex adjustment did not materially attenuate the cluster effect for most metabolic outcomes. For serum uric acid, the cluster effect remained highly significant (*p* = 1.94 × 10^−5^ vs. 1.70 × 10^−5^; Δ*R* ^2^ = +0.016), as for ALT, total hip T‐score and BUN (all Δ*R* ^2^ ≤ 0.016) (Tables S2 and S3), indicating sex contributed minimally to cluster‐explained variance for these outcomes.

Within‐sex analyses supported this conclusion (Figure 2D). Among males (Clusters 1, 2, 4; Cluster 3 had one male), ALT, total hip T‐score, BUN and triglycerides differed significantly after correction. Among females (Clusters 1, 3, 4; Cluster 2 had one female), total hip *T*‐ and *Z*‐scores remained significant, with a HOMA‐IR trend. However, significant sex‐by‐cluster interactions were detected for hip *T*‐score (*p* = 0.003) and hip *Z*‐score (*p* = 0.014), suggesting sex‐dependent cluster effects on bone density.

Collectively, sex is a major determinant of body composition and Clusters 2 and 3 are strongly sex‐differentiated, body‐composition phenotypes carry partially independent metabolic information within each sex stratum. For outcomes with significant sex‐by‐cluster interactions (Figure 2C), associations should be interpreted with recognition of this sex dependence.

Under sex‐specific standardization, the cluster–sex association was eliminated (ARI = 0.002 vs. 0.378 for the original clustering) and agreement with the original four‐cluster structure was moderate (ARI = 0.351), Nevertheless, the sex‐standardized four‐cluster solution retained a central‐adiposity cluster (highest A/G ratio, 1.3) and remained associated with all key outcomes (ALT, γ‐GGT, hip *T*‐score, BUN, HDL‐C, triglycerides, HOMA‐IR), as well as osteoporosis (*p* = 0.003), MASLD (*p* < 0.001) and hyperuricemia (*p* < 0.001) (Table S9, Figure S1). These findings indicate that although sex‐related differences substantially contributed to the clustering structure, residual body‐composition heterogeneity remained associated with metabolic traits and complications.

The number of clusters was chosen by silhouette width (males: 0.325 [*k* = 2] vs. 0.257 [*k* = 3]; females: 0.327 [*k* = 2] vs. 0.250 [*k* = 3]) (Table S11). Sex‐stratified clustering within males (*k* = 3) distinguished a low‐fat/lean phenotype, an intermediate phenotype with the highest A/G ratio (1.34) and a central‐obesity/high‐muscle phenotype, which differed in ALT, γ‐GGT, uric acid, hip T‐score, BUN, HDL‐C, triglycerides, HOMA‐IR, MASLD and hyperuricemia (all *p* < 0.05). Within females (*k* = 2), a lean/low‐adiposity phenotype differed from a high‐adiposity phenotype in ALT, γ‐GGT, uric acid, hip *T*‐score, HOMA‐IR and osteoporosis (all *p* < 0.05) (Table S11).

### 3.3 Body Composition Characteristics

The four clusters exhibited substantial heterogeneity in fat/lean distribution (Table S1, Figure 1B,C). Because body‐composition variables constituted the clustering inputs, formal statistical comparisons across clusters are circular and are presented descriptively. Integrating AWGS criteria and established adiposity parameters [12], the clusters were characterized as follows:

Cluster 1 (Low Fat‐Low Lean Mass, *n* = 42, 66.7% male) had the lowest total, lean and fat mass, with ASMI below AWGS thresholds in both sexes and elevated body‐fat percentage and A/G ratio.

Cluster 2 (Male‐Predominant Central Obesity, *n* = 71, 98.6% male) had preserved muscle mass (ASMI within normal limits) but elevated fat percentage and the highest A/G ratio, indicating centripetal adiposity; near‐complete male predominance precludes separating cluster‐specific from sex effects.

Cluster 3 (Female‐Predominant Low‐Muscle High‐Adiposity, *n* = 49, 98.0% female) showed high adiposity with relatively low lean mass, a pattern resembling a low‐muscle high‐adiposity phenotype reported in previous studies; muscle function was not assessed, so this does not equate to a clinical sarcopenia diagnosis. As with Cluster 2, near‐complete female predominance precludes separating cluster‐specific from sex effects.

Cluster 4 (High Fat‐High Lean Mass, *n* = 23, 65.2% male) had the highest total mass, fat mass, lean mass and VAT, associated with several adverse markers.

### 3.4 Glycemic Profiles and Pancreatic Function

Glycemic parameters did not differ across clusters (HbA1c, FPG, post‐load glucose, HOMA‐β; all adjusted *p* = 1.000) (Table S1, Figure 3A,B). HOMA‐IR showed a trend that did not survive correction (raw *p* < 0.001; adjusted *p* = 0.055). Although fasting and early post‐load insulin and C‐peptide were mostly within reference ranges, peak secretion was delayed beyond 60 min in all clusters; Clusters 3 and 4 showed the highest, and Cluster 1 the lowest, exposure—patterns that remain descriptive given the non‐significant HOMA‐IR.

**FIGURE 3:**
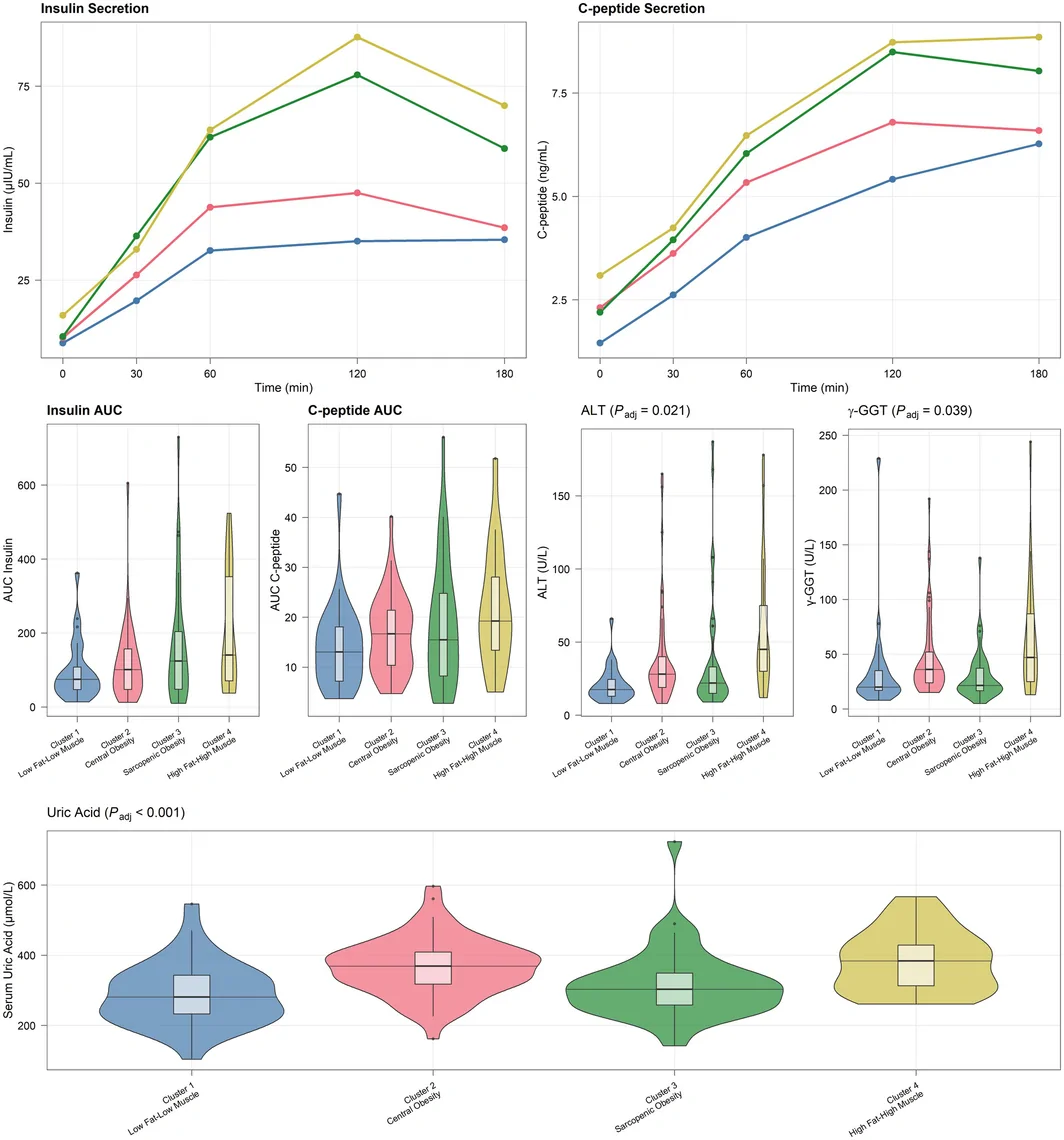
Metabolic profiles and secretory dynamics across body composition clusters. (A) Insulin (left) and C‐peptide (right) secretion curves during the 75‐g OGTT. Mean concentrations plotted at 0, 30, 60, 120 and 180 min. Shaded bands represent ± SEM. Cluster 4 exhibits a pronounced hyperinsulinemic profile; Clusters 2–4 show delayed peak secretion beyond 60 min; Cluster 1 displays a blunted curve without a distinct peak. (B) Area under the curve (AUC) for insulin (left) and C‐peptide (right). Violin plots with overlaid boxplots. Cluster 4 shows the highest AUC for both; Cluster 1 the lowest. Neither HOMA‐IR (adjusted *p* = 0.055) nor AUC values retained significance after Bonferroni correction. (C) ALT (left, adjusted *p* = 0.021) and gamma‐GGT (right, adjusted *p* = 0.039) across clusters. Both retained significance after Bonferroni correction. Cluster 4 exhibits the highest levels. AST did not retain significance (adjusted *p* = 1.000). (D) Serum uric acid concentrations across clusters (adjusted *p* < 0.001). Clusters 2 and 4 show elevated SUA. ALT, alanine aminotransferase; GGT, gamma‐glutamyl transferase; OGTT, oral glucose tolerance test; SUA, serum uric acid.

### 3.5 Hepatic Enzyme Profiles

ALT (adjusted *p* = 0.021) and γ‐GGT (adjusted *p* = 0.039) differed across clusters, highest in Cluster 4; AST did not retain significance (adjusted *p* = 1.000). Cluster 2 and Cluster 4 showed higher γ‐GGT than Clusters 1 and 3 (Figure 3C), suggesting visceral adiposity‐predominant clusters carry elevated hepatic and cholestatic markers.

### 3.6 Renal Function Parameters

Serum creatinine (SCr) and BUN differed across clusters (adjusted *p* < 0.001 and 0.031, respectively), lowest in Cluster 3—consistent with its low lean mass—whereas intrinsic renal function (eGFR, ACR, UAER) did not differ (all adjusted *p* = 1.000). After adjustment (Table S5), obesity‐related clusters showed lower BUN than Cluster 1.

### 3.7 Bone Mineral Density and Bone Turnover Markers

Lumbar spine *T*‐/*Z*‐scores did not differ across clusters (adjusted *p* = 1.000). Hip and femoral‐neck BMD showed heterogeneity (Table S1, Figure 4A), with significant differences persisting after correction for left total hip T‐score (*p* < 0.001), left total hip *Z*‐score (*p* = 0.008), left neck *T*‐score (*p* = 0.009), right total hip *T*‐score (*p* = 0.010), right total hip *Z*‐score (*p* = 0.043) and right neck *T*‐score (*p* = 0.034). Cluster 4 had the highest and Cluster 1 the lowest hip BMD, possibly consistent with differential mechanical loading from muscle mass.

**FIGURE 4:**
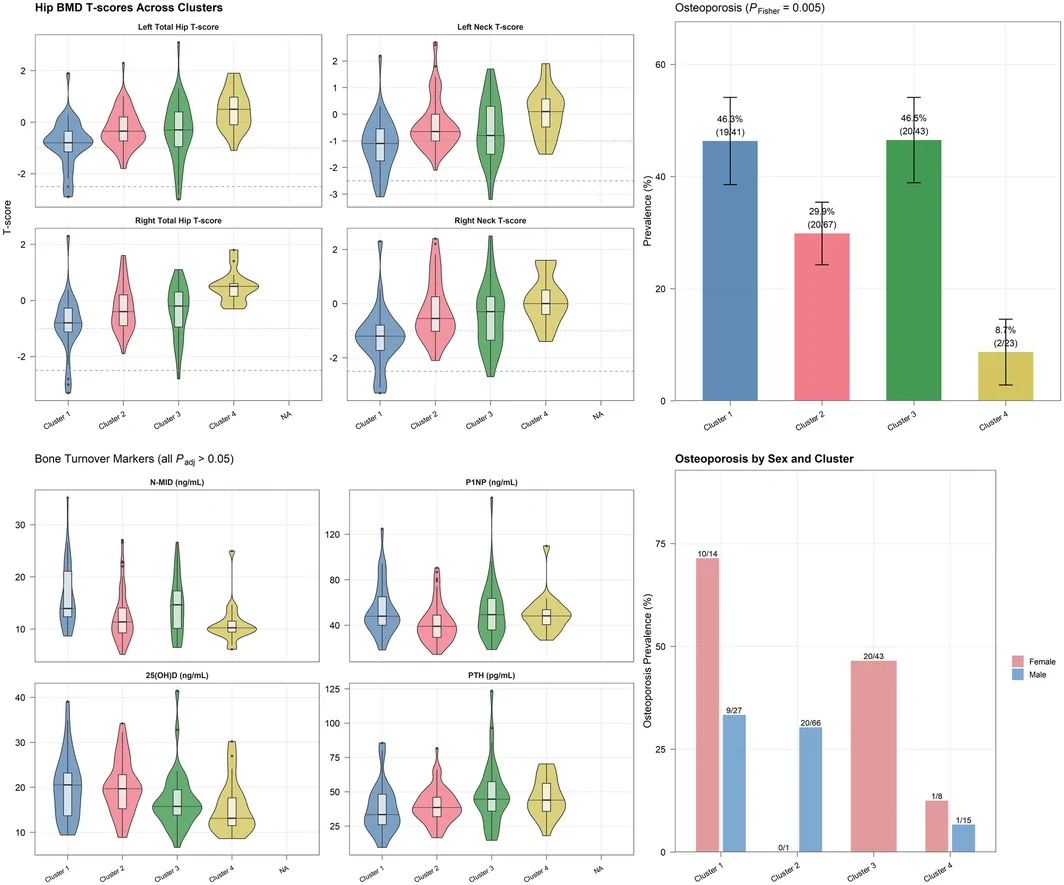
Bone mineral density, osteoporosis prevalence and bone turnover markers across body composition clusters. (A) Hip BMD *T*‐scores at four sites: Left total hip (adjusted *p* < 0.001), left femoral neck (adjusted *p* = 0.009), right total hip (adjusted *p* = 0.010), right femoral neck (adjusted *p* = 0.034). Grey dotted line: Osteopenia threshold (*T* = −1.0); grey dashed line: Osteoporosis threshold (*T* = −2.5). Cluster 4 shows the highest BMD; Cluster 1 the lowest. Lumbar spine T‐scores did not differ (adjusted *p* = 1.000). (B) Osteoporosis prevalence by cluster. Numbers above bars: Cases/total with known status. Fisher exact *p* = 0.005. (C) Bone turnover markers across clusters. N‐MID osteocalcin, P1NP, 25(OH)D and PTH. All adjusted *p* > 0.05 after Bonferroni correction; differences should not be interpreted as robust biological signals. (D) Osteoporosis prevalence stratified by sex and cluster. 25(OH)D, 25‐hydroxyvitamin D; BMD, bone mineral density; BTMs, bone turnover markers; N‐MID, N‐terminal mid‐fragment osteocalcin; P1NP, procollagen Type 1 N‐terminal propeptide; PTH, parathyroid hormone.

No bone turnover marker (BTM) retained significance after Bonferroni correction: N‐MID (*p* = 0.100), P1NP (*p* = 1.000), 25(OH)D (*p* = 0.599) and PTH (*p* = 1.000), indicating cluster membership does not independently discriminate bone turnover status and that unadjusted associations likely reflect multiple testing rather than robust biological signals (Figure 4C). Hip BMD differences, however, appear robust to correction and likely reflect mechanical and endocrine influences of muscle and fat mass; a significant sex‐by‐cluster interaction for hip *T*‐score (*p* = 0.003) further suggests sex‐dependent bone effects.

### 3.8 Serum Uric Acid Level

Serum uric acid (SUA) differed across clusters (adjusted *p* < 0.001), highest in Clusters 4 and 2, which are characterized by greater lean mass and adiposity. After adjustment for age, sex, smoking and alcohol consumption (Table S5, Figure 3D), Cluster 4 showed the strongest association (β = 72, 95% CI 22–123, *p* = 0.005), with Cluster 2 (β = 61, *p* = 0.003) and Cluster 3 (β = 59, *p* = 0.036) also elevated; these associations were robust to sex adjustment.

### 3.9 Associations With Chronic Complications and Comorbidities

Complication definitions were: DKD (UACR ≥ 30 mg/g or eGFR < 60 mL/min/1.73 m^2^ persisting > 3 months); DR (fundus examination by an experienced ophthalmologist); LEAD (lower‐extremity ultrasound showing arteriosclerosis); MASLD (liver ultrasound); CAD (coronary CT angiography, prior angiography/CTCA, or stent history); and HTN (BP > 140/90 mmHg on two occasions, prior diagnosis, or antihypertensive use).

A comprehensive missing data report is provided in Table S6. Missingness ranged from 0% (hyperuricemia) to 43.5% (DR); complete‐case models retained 96 (DR) to 156 (osteoporosis) of 186 participants.

In univariate analyses of known cases, cluster membership was associated with osteoporosis (Fisher's exact *p* = 0.005), MASLD (*p* < 0.001) and hyperuricemia (*p* = 0.020), with a borderline LEAD trend (*p* = 0.053). DKD, DR, carotid plaque, HTN, CAD and dyslipidemia showed no significant associations (all *p* > 0.05; Table S6).

Table S12 presents pooled results from multiple‐imputation‐adjusted logistic regression models for the three outcomes with significant univariate associations; complete‐case sensitivity analyses were directionally consistent (Table S7).

For osteoporosis, Cluster 4 had lower odds than Cluster 1 (OR = 0.16, 95% CI 0.03–0.92, *p* = 0.040); age (OR = 1.07/year, *p* < 0.001), male sex (OR = 0.14, *p* = 0.013) and smoking (OR = 5.30, *p* = 0.005) were independent predictors (Table S12, Figure 5, Figure 4B).

**FIGURE 5:**
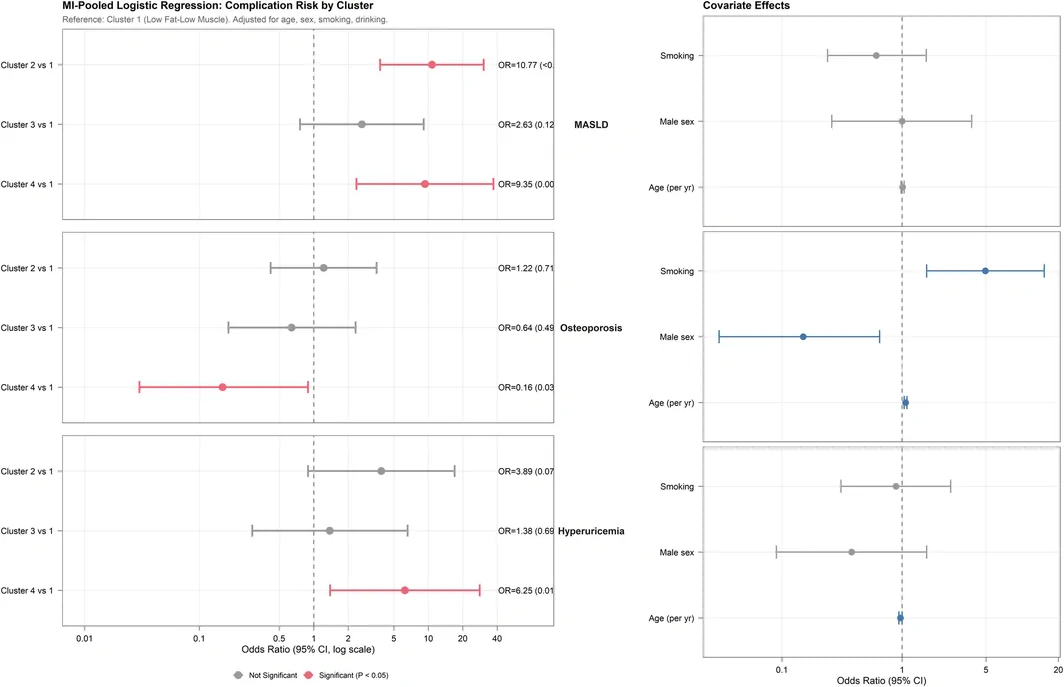
Multiple imputation‐pooled logistic regression analysis of clinical complication risk across body composition clusters. (Left) Cluster‐specific odds ratios for MASLD, osteoporosis and hyperuricemia. ORs were estimated from logistic regression models fitted to 20 MICE datasets and pooled using Rubin rules. Cluster 1 (Low Fat‐Low Muscle) is the reference group. All models adjusted for age, sex, smoking and alcohol consumption. Red circles: *p* < 0.05; grey circles: *p* ≥ 0.05. Error bars denote 95% CIs (log scale). Dashed vertical line at OR = 1. MASLD: Cluster 2 (OR = 9.69, *p* < 0.001) and Cluster 4 (OR = 8.55, *p* = 0.002) show markedly elevated odds. Osteoporosis: Cluster 4 is protective (OR = 0.16, *p* = 0.040). Hyperuricemia: Cluster 4 shows elevated odds (OR = 6.36, *p* = 0.016). (Right) Covariate effects from the same MI‐pooled models. Blue circles: *p* < 0.05; grey circles: *p* ≥ 0.05. Age (OR = 1.07/year, *p* < 0.001), male sex (OR = 0.14, *p* = 0.013) and smoking (OR = 5.30, *p* = 0.005) were independently associated with osteoporosis risk. CI, confidence interval; MASLD, metabolic dysfunction‐associated steatotic liver disease; MICE, multiple imputation by chained equations; OR, odds ratio.

For MASLD, Clusters 2 and 4 had markedly higher odds versus Cluster 1 (OR = 9.69, 95% CI 3.34–28.09, *p* < 0.001; and OR = 8.55, 95% CI 2.18–33.51, *p* = 0.002), whereas Cluster 3 did not (OR = 2.44, *p* = 0.142); age, sex, smoking and drinking were non‐significant.

For hyperuricemia, Cluster 4 had higher odds (OR = 6.36, 95% CI 1.42–28.57, *p* = 0.016), whereas Cluster 2 did not reach significance (OR = 3.99, *p* = 0.066); age was inversely associated (OR = 0.97/year, *p* = 0.049) (Table S12, Figure 5).

For LEAD, only age remained significant (OR = 1.14, *p* < 0.001), with no independent cluster effects; DKD and DR also showed no cluster effects in adjusted models. The DR analysis was severely limited by 43.5% missingness (8 confirmed cases among 105 with known status), precluding meaningful inference (Table S7).

To assess incremental utility beyond conventional measures, we compared nested models using three predictors: BMI, BMI plus waist circumference (WC) and BMI plus visceral adipose tissue area (VAT). Cluster membership added significant incremental value for MASLD (Δ McFadden pseudo‐*R* ^2^ 0.064–0.076; likelihood‐ratio test *p* = 0.003–0.009) and BUN (Δ*R* ^2^ = 0.050–0.073; F‐test *p* = 0.014–0.067), reflecting information not captured by these indices. These increments persisted after adjusting for VAT, indicating they did not merely reflect the VAT component. For all other outcomes, the cluster added no significant incremental value (all *p* > 0.05) (Table S10). In this treatment‐naive cohort, incremental clinical utility was limited.

## 4 Discussion

This study identified four body‐composition patterns with distinct profiles. Variance decomposition showed cluster assignments capture information beyond sex for fat distribution. However, the near‐complete sex segregation of Clusters 2 and 3 and the modest silhouette width (0.272) indicate these are a pragmatic descriptive framework rather than discrete sex‐independent categories. After multiple imputation, associations were confirmed for MASLD, osteoporosis and hyperuricemia, but not DKD, DR, or LEAD.

### 4.1 Comparison With Existing T2DM Subtyping Frameworks

Our body‐composition phenotypes partially overlap with previously reported clinical diabetes subgroups but provide additional anatomical information. The ANDIS subtypes [2] did not incorporate body composition; our DXA approach adds anatomical information—Cluster 1 resembles a MARD–SIDD spectrum, whereas Clusters 2 and 4 partially overlap SIRD and MOD. Dennis's two‐axis model [3] is extended by our suggestion that muscle–fat balance represents a contributory axis. Head‐to‐head comparisons would be needed to establish incremental clinical utility.

Our findings should also be interpreted against body‐composition studies in other populations. The predominance of low‐muscle high‐adiposity among women and central obesity among men in our Chinese cohort is consistent with East Asian reports of higher sarcopenia burden in older diabetic women [12]. Western cohorts show a more pronounced ‘high fat–high lean’ phenotype with greater absolute lean mass, reflecting ethnic differences in muscle mass and adiposity. Our osteoporosis prevalence (35.1%) is comparable to or slightly higher than Western T2DM cohorts, possibly reflecting the older age and lower BMI of Cluster 1. Body‐composition subtypes are therefore population‐dependent and may not translate to non‐Asian cohorts; cross‐population validation is needed.

### 4.2 Phenotypic Characteristics and the Sex‐Cluster Relationship

A central finding is the strong sex differentiation of the clusters: Cluster 2 is 98.6% male and Cluster 3 is 98.0% female, so cluster membership and sex are statistically non‐separable for these two groups. Sex‐specific standardization eliminated the cluster–sex association (ARI = 0.002) but showed moderate agreement with the original clustering (ARI = 0.351), confirming the original clustering was substantially driven by sex‐related magnitude differences; nevertheless, the sex‐standardized solution retained a central‐adiposity cluster and remained associated with all examined outcomes (Figure 2, Table S9). Re‐clustering with 30 less‐correlated variables reproduced the four‐cluster structure (ARI = 0.912), further supporting robustness.

We therefore designate Cluster 2 ‘Male‐Predominant Central Obesity’ and Cluster 3 ‘Female‐predominant low‐muscle high‐adiposity’ (a low‐muscle high‐adiposity phenotype), while acknowledging that these labels partly conflate sex with body‐composition pattern. The sex‐standardized analysis suggests the central‐adiposity pattern may represent a genuine, sex‐independent phenotype. Sex‐stratified clustering within males identified central‐obesity and low‐muscle phenotypes, whereas within females it distinguished high‐adiposity from lean phenotypes; the absence of a discrete high‐fat/low‐muscle subgroup within females may partly reflect the smaller female sample (*n* = 71) rather than the absence of this phenotype, and larger sex‐stratified studies are needed to determine whether this phenotype emerges in females.

### 4.3 Cluster‐Specific Metabolic Profiles

Cluster 1 comprised older individuals with lean and fat depletion and the highest osteoporosis prevalence (46.3%), possibly reflecting reduced mechanical loading and myokine‐mediated bone formation [13, 14]; the cross‐sectional design precludes causal inference (Figure 4A,B).

Cluster 2, with substantial visceral adiposity and the highest A/G ratio, showed the strongest MASLD association (OR = 9.69), consistent with the pathogenic role of visceral adiposity in hepatic steatosis [15, 16] and a trend toward hyperuricemia (Figure 5).

Cluster 3 showed a ‘high‐fat, low‐muscle’ imbalance with unremarkable hepatic profiles; it did not differ from Cluster 1 for MASLD (OR = 2.44) or osteoporosis (OR = 0.65), suggesting a distinct risk profile warranting investigation.

Cluster 4, the youngest subtype, carried the greatest adverse metabolic burden—highest MASLD (OR = 8.55) and hyperuricemia (OR = 6.36) but lowest osteoporosis odds (OR = 0.16)—suggesting a metabolic ‘trade‐off’ in which greater lean mass may protect bone despite high adiposity, consistent with visceral adiposity–purine metabolism links [17, 18, 19, 20, 21, 22] (Table S12, Figure 5).

### 4.4 Missing Data and Analytical Limitations

A critical consideration is the extent of missing data. DR (43.5%), carotid plaque (41.4%) and LEAD (31.7%) were frequently unknown; the DR analysis (8 cases among 105) precludes inference. Multiple imputation addressed missingness, and complete‐case/MI consistency for MASLD, osteoporosis and hyperuricemia supports robustness. However, the missing‐at‐random assumption cannot be verified, and non‐random missingness could bias MI estimates (Tables S6 and S8).

Additional limitations include: the single‐center, cross‐sectional design, which precludes causal or temporal inference between body‐composition subtypes and complications; the modest silhouette width (0.272), indicating a continuum rather than discrete clusters, which may partly reflect the high‐dimensional clustering approach that reduced cluster separation; the modest sample size (*n* = 185) for clustering with 50 variables, with a small Cluster 4 (*n* = 23) limiting estimate precision; lack of external validation in an independent cohort; sex‐cluster non‐separability for Clusters 2 and 3; and reliance on ultrasound rather than biopsy for MASLD, introducing possible misclassification.

### 4.5 Clinical Implications

Despite these limitations, the association of body‐composition patterns with differential complication risks—even after sex adjustment and multiple imputation—has potential clinical relevance. The elevated MASLD odds in high‐adiposity clusters (2 and 4) suggest DXA‐based assessment at diagnosis may help characterize individuals considered for further hepatic steatosis assessment, while the elevated osteoporosis risk in the low‐muscle phenotype (Cluster 1) supports body‐composition assessment for fracture‐risk stratification. However, compared with conventional indices (BMI, BMI plus waist circumference and BMI plus visceral adipose tissue), the clustering framework added significant incremental predictive value only for MASLD and BUN—and this increment persisted even after adjusting for VAT—but not for other complications or metabolic indices (Table S10). This limited incremental value is expected in this treatment‐naive, newly diagnosed T2DM cohort, where overt complications are uncommon and the cross‐sectional design cannot capture prognostic trajectories. Future prospective, multicenter cohorts with systematic complication ascertainment and extended follow‐up may reveal greater incremental clinical utility of body‐composition subtyping for stratification (Table S12, Figure 5).

## 5 Conclusion

In treatment‐naive T2DM, DXA‐derived body composition identifies four internally stable clustering patterns associated with distinct metabolic and comorbidity profiles: visceral‐adiposity clusters (2 and 4) carry the highest MASLD and hyperuricemia risk, whereas the low‐muscle‐mass phenotype (Cluster 1) carries the highest osteoporosis risk. These associations remained detectable after sex‐specific normalization and multiple imputation, though the clusters substantially overlap with sex‐based differences and the modest silhouette width indicates body composition is better viewed as a continuum. DXA‐based profiling may aid complication‐risk stratification, but prospective multicenter validation is required.

## Author Contributions

**Xintong Zhang:** investigation, methodology, software, formal analysis, data curation, visualization, writing – original draft, writing – review and editing. **Rui Zhang:** investigation, data curation. **Tao Jiang:** conceptualization, funding acquisition, writing – review and editing, resources, supervision, project administration.

## Funding

The authors declare that this study, including manuscript preparation and publication (covering Rapid Service Fees), was supported by grants from the Project of Science and Technology Research and Development of China Railway Corporation [Grant number: J2021Z608].

## Ethics Statement

This research was carried out following the guidelines set out in the Declaration of Helsinki and the study protocol was approved by the Ethics Committee of Beijing Shijitan Hospital, Capital Medical University (Approval Number: sjtkyll‐lx‐2021(27)). Written informed consent was obtained from all participants.

## Conflicts of Interest

The authors declare no conflicts of interest.

## Supporting information


**Figure S1:** Sex‐specific standardization sensitivity analysis of the body‐composition clusters. (A) Sex distribution across clusters under whole‐cohort *Z*‐score standardization (the original approach); two clusters show near‐complete sex segregation (Cluster 2: 98.6% male; Cluster 3: 98.0% female). (B) Sex distribution across clusters after sex‐specific standardization (each DXA variable *Z*‐scored within sex before pooling); the cluster–sex association was eliminated. (C) Adjusted Rand Index (ARI) quantifying agreement: ARI (original clusters vs. sex) = 0.378; ARI (sex‐specific clusters vs. sex) = 0.002; ARI (original vs. sex‐specific clusters) = 0.351. Only 35.1% of the original cluster structure was preserved after sex‐specific standardization, indicating that the original four‐cluster structure was substantially driven by sex‐related magnitude differences; nevertheless, cluster–outcome associations remained detectable after sex‐specific normalization. ARI, adjusted Rand index; DXA, dual‐energy X‐ray absorptiometry.

**Table S1:** Baseline characteristics, body composition and metabolic profiles of treatment‐naive T2DM patients stratified by body composition cluster (*N* = 185).

**Table S2:** Sex‐stratified Kruskal–Wallis tests and sex‐by‐cluster interaction analyses for key metabolic outcomes.

**Table S3:** Nested model comparison: variance explained by cluster alone (Model 1) vs. cluster + sex (Model 2) for key metabolic outcomes.

**Table S4:** Variance decomposition: unique contributions of cluster membership and sex to DXA‐derived body composition variables.

**Table S5:** Multivariable linear regression: associations of body composition clusters with metabolic outcomes.

**Table S6:** Distribution of chronic complications and comorbidities across body composition clusters (corrected).

**Table S7:** Sensitivity analysis: complete‐case vs. multiple imputation logistic regression for complication outcomes.

**Table S8:** Missing data report for variables used in complication and regression analyses (*N* = 186).

**Table S9:** Influence of sex on cluster structure and outcome associations: whole‐cohort vs. sex‐specific standardization (50 DXA variables).

**Table S10:** Incremental clinical utility of the clustering framework beyond conventional indices (BMI, BMI + WC and BMI + VAT): nested model comparison.

**Table S11:** Sex‐stratified clustering: within‐sex body‐composition phenotypes, k‐selection and metabolic associations.

**Table S12:** Multiple imputation‐pooled logistic regression analysis of clinical complication risk across body composition clusters.

## Data Availability

The data that support the findings of this study are available on request from the corresponding author. The data are not publicly available due to privacy or ethical restrictions.
