# Supplementary material for: Body Composition Phenotypes and Their Metabolic Associations in Treatment‐Naïve Patients With Newly Diagnosed Type 2 Diabetes: A DXA‐Based Cluster Analysis
Source: Endocrinol Diabetes Metab. 2026 Oct 19;9(6):e70366. doi: 10.1002/edm2.70366 (PMC13680683; doi:10.1002/edm2.70366)
Supplement: Supplementary file 1 [file EDM2-9-e70366-s001.docx]

# Table 1**2.** Multiple Imputation-Pooled Logistic Regression Analysis of Clinical Complication Risk Across Body Composition Clusters

| **Outcome** | **Predictor** | **OR** | **95% CI** | **P-value** |
| --- | --- | --- | --- | --- |
| **Osteoporosis** | **Intercept** | **0.04** | **0.00–0.30** | **0.0018** |
|  | Cluster 2 vs 1 | 1.23 | 0.42–3.61 | 0.7076 |
|  | Cluster 3 vs 1 | 0.65 | 0.18–2.34 | 0.5068 |
|  | **Cluster 4 vs 1** | **0.16** | **0.03–0.92** | **0.0395** |
|  | **Age (per year)** | **1.07** | **1.04–1.10** | **<0.001** |
|  | **Male sex (ref: Female)** | **0.14** | **0.03–0.66** | **0.0131** |
|  | **Current smoker (ref: Non-smoker)** | **5.30** | **1.68–16.76** | **0.0046** |
|  | Current drinker (ref: Non-drinker) | 0.62 | 0.23–1.69 | 0.3471 |
| MASLD (NAFLD) | Intercept | 0.37 | 0.06–2.34 | 0.2895 |
|  | **Cluster 2 vs 1** | **9.69** | **3.34–28.09** | **<0.001** |
|  | Cluster 3 vs 1 | 2.44 | 0.74–8.02 | 0.1418 |
|  | **Cluster 4 vs 1** | **8.55** | **2.18–33.51** | **0.0021** |
|  | Age (per year) | 1.01 | 0.98–1.03 | 0.6529 |
|  | Male sex (ref: Female) | 1.02 | 0.28–3.67 | 0.9778 |
|  | Current smoker (ref: Non-smoker) | 0.62 | 0.23–1.67 | 0.3433 |
|  | Current drinker (ref: Non-drinker) | 1.57 | 0.54–4.52 | 0.4085 |
| Hyperuricemia | Intercept | 0.66 | 0.08–5.51 | 0.7012 |
|  | Cluster 2 vs 1 | 3.99 | 0.91–17.40 | 0.0659 |
|  | Cluster 3 vs 1 | 1.40 | 0.29–6.71 | 0.6734 |
|  | **Cluster 4 vs 1** | **6.36** | **1.42–28.57** | **0.0158** |
|  | **Age (per year)** | **0.97** | **0.94–1.00** | **0.0489** |
|  | Male sex (ref: Female) | 0.37 | 0.09–1.57 | 0.1789 |
|  | Current smoker (ref: Non-smoker) | 0.86 | 0.31–2.40 | 0.7715 |
|  | Current drinker (ref: Non-drinker) | 1.14 | 0.41–3.18 | 0.8020 |
| MI: 20 imputed datasets (MICE), pooled by Rubin's rules. All models adjusted for age, sex, smoking, and alcohol consumption. Reference group: Cluster 1 (Low Fat-Low Muscle). Bold rows indicate statistically significant associations (P<0.05). | | | | |
