# Supplementary material for: Body Composition Phenotypes and Their Metabolic Associations in Treatment‐Naïve Patients With Newly Diagnosed Type 2 Diabetes: A DXA‐Based Cluster Analysis
Source: Endocrinol Diabetes Metab. 2026 Oct 19;9(6):e70366. doi: 10.1002/edm2.70366 (PMC13680683; doi:10.1002/edm2.70366)
Supplement: Supplementary file 2 [file EDM2-9-e70366-s002.docx]

# Supplemental Table S9. Influence of sex on cluster structure and outcome associations: whole-cohort vs sex-specific standardization (50 DXA variables).

## A. Sex distribution by cluster — original (whole-cohort) clustering (Table S1)

| **Sex** | **Cluster 1** | **Cluster 2** | **Cluster 3** | **Cluster 4** |
| --- | --- | --- | --- | --- |
| Female | 14 | 1 | 48 | 8 |
| Male | 28 | 70 | 1 | 15 |

## B. Sex distribution by cluster — sex-specific standardization (k=4)

| **Sex** | **Cluster 1** | **Cluster 2** | **Cluster 3** | **Cluster 4** |
| --- | --- | --- | --- | --- |
| Female | 11 | 21 | 21 | 18 |
| Male | 14 | 28 | 43 | 29 |

## C. Agreement and separation metrics

| **Metric** | **Value** |
| --- | --- |
| ARI (original clusters vs sex) | 0.378 |
| ARI (sex-specific clusters vs sex) | 0.002 |
| ARI (original vs sex-specific clusters) | 0.351 |
| Silhouette width, whole-cohort k=4 | 0.283 |
| Silhouette width, sex-specific k=4 | 0.178 |

## D. k-selection under sex-specific standardization

| **k** | **sil** | **CH** |
| --- | --- | --- |
| 2 | 0.330 | 110.5 |
| 3 | 0.238 | 91.7 |
| 4 | 0.178 | 76.4 |
| 5 | 0.183 | 68.1 |
| 6 | 0.177 | 60.7 |

## E. Outcome associations: original vs sex-specific k=4 clustering

| **Outcome** | **Original P** | **Sex-specific P** | **Retained?** |
| --- | --- | --- | --- |
| ALT, U/L | <0.001 | <0.001 | Yes |
| γ-GGT, U/L | <0.001 | <0.001 | Yes |
| UA, μmol/L | <0.001 | <0.001 | Yes |
| Total hip T-score | <0.001 | <0.001 | Yes |
| BUN, mmol/L | <0.001 | 0.0382 | Yes |
| HDL-C, mmol/L | <0.001 | 0.0071 | Yes |
| TG, mmol/L | <0.001 | <0.001 | Yes |
| HOMA-IR | <0.001 | <0.001 | Yes |

### Complication outcomes (Fisher exact test):

| **Complication** | **Original P** | **Sex-specific P** | **Retained?** |
| --- | --- | --- | --- |
| Osteoporosis | 0.0050 | 0.0028 | Yes |
| MASLD (NAFLD) | <0.001 | <0.001 | Yes |
| Hyperuricemia | 0.0207 | <0.001 | Yes |

Methods: 185 participants with complete data on 50 DXA body-composition variables (LArmFatMassg to AppenLeanBMCHt2kgm2), matching the primary analysis. Whole-cohort standardization Z-scored variables across the entire cohort; sex-specific standardization Z-scored within each sex before pooling. Both used k-means (k=4, nstart=50). The original clustering used the author's cluster labels (Table S1); sex-specific clustering was re-derived for this sensitivity analysis. ARI = Adjusted Rand Index (0 = chance, 1 = perfect). Key finding: sex-specific standardization eliminated the cluster–sex correlation and preserved only a fraction of the original structure, indicating the original four-cluster structure was partly driven by sex-related magnitude differences; nevertheless, cluster–outcome associations remained significant, indicating these associations are not attributable to sex-related magnitude alone.
