# Supplementary material for: Body Composition Phenotypes and Their Metabolic Associations in Treatment‐Naïve Patients With Newly Diagnosed Type 2 Diabetes: A DXA‐Based Cluster Analysis
Source: Endocrinol Diabetes Metab. 2026 Oct 19;9(6):e70366. doi: 10.1002/edm2.70366 (PMC13680683; doi:10.1002/edm2.70366)
Supplement: Supplementary file 3 [file EDM2-9-e70366-s003.docx]

# Supplemental Table S5. Multivariable linear regression: associations of body composition clusters with metabolic outcomes.

| **Outcome** | **Predictor** | **Beta** | **95% CI** | **P-value** |
| --- | --- | --- | --- | --- |
| **ALT, U/L** |  |  |  |  |
|  | Cluster 2 vs 1 | 17.34 | -0.30 to 34.99 | 0.0563 |
|  | Cluster 3 vs 1 | 4.89 | -18.61 to 28.38 | 0.6843 |
|  | Cluster 4 vs 1 | 29.24 | 8.18 to 50.31 | 0.0075 |
|  | Age (per year) | -0.55 | -0.98 to -0.11 | 0.0145 |
|  | Male sex | -21.94 | -44.33 to 0.45 | 0.0572 |
|  | Current smoking | 3.04 | -12.73 to 18.81 | 0.7062 |
|  | Current drinking | 11.90 | -4.00 to 27.80 | 0.1450 |
| **AST, U/L** |  |  |  |  |
|  | Cluster 2 vs 1 | 5.01 | -7.36 to 17.39 | 0.4288 |
|  | Cluster 3 vs 1 | 1.49 | -15.07 to 18.06 | 0.8600 |
|  | Cluster 4 vs 1 | 17.17 | 2.32 to 32.02 | 0.0252 |
|  | Age (per year) | -0.23 | -0.54 to 0.07 | 0.1353 |
|  | Male sex | -15.21 | -30.96 to 0.54 | 0.0607 |
|  | Current smoking | 3.18 | -7.88 to 14.23 | 0.5740 |
|  | Current drinking | 8.94 | -2.23 to 20.10 | 0.1193 |
| **γ-GGT, U/L** |  |  |  |  |
|  | Cluster 2 vs 1 | 13.10 | -4.17 to 30.38 | 0.1397 |
|  | Cluster 3 vs 1 | -1.48 | -24.21 to 21.25 | 0.8986 |
|  | Cluster 4 vs 1 | 28.24 | 7.70 to 48.78 | 0.0080 |
|  | Age (per year) | -0.46 | -0.89 to -0.04 | 0.0346 |
|  | Male sex | -12.15 | -33.61 to 9.32 | 0.2695 |
|  | Current smoking | 9.77 | -5.52 to 25.06 | 0.2128 |
|  | Current drinking | 19.61 | 4.23 to 34.99 | 0.0138 |
| **UA, μmol/L** |  |  |  |  |
|  | Cluster 2 vs 1 | 60.68 | 20.81 to 100.54 | 0.0034 |
|  | Cluster 3 vs 1 | 59.32 | 4.44 to 114.19 | 0.0359 |
|  | Cluster 4 vs 1 | 72.46 | 22.21 to 122.70 | 0.0054 |
|  | Age (per year) | -1.11 | -2.16 to -0.06 | 0.0401 |
|  | Male sex | 37.37 | -15.79 to 90.54 | 0.1705 |
|  | Current smoking | -1.20 | -37.29 to 34.88 | 0.9480 |
|  | Current drinking | 16.04 | -20.14 to 52.22 | 0.3864 |
| **BUN, mmol/L** |  |  |  |  |
|  | Cluster 2 vs 1 | -1.06 | -1.78 to -0.35 | 0.0041 |
|  | Cluster 3 vs 1 | -1.03 | -2.01 to -0.06 | 0.0396 |
|  | Cluster 4 vs 1 | -1.51 | -2.35 to -0.68 | <0.001 |
|  | Age (per year) | 0.03 | 0.01 to 0.05 | 0.0026 |
|  | Male sex | 0.44 | -0.50 to 1.38 | 0.3632 |
|  | Current smoking | 0.37 | -0.28 to 1.02 | 0.2671 |
|  | Current drinking | -0.00 | -0.65 to 0.65 | 0.9959 |
| **Cr, μmol/L** |  |  |  |  |
|  | Cluster 2 vs 1 | -0.35 | -7.03 to 6.34 | 0.9195 |
|  | Cluster 3 vs 1 | 9.31 | 0.11 to 18.51 | 0.0493 |
|  | Cluster 4 vs 1 | 3.16 | -5.27 to 11.58 | 0.4642 |
|  | Age (per year) | 0.23 | 0.05 to 0.40 | 0.0128 |
|  | Male sex | 23.99 | 15.08 to 32.91 | <0.001 |
|  | Current smoking | -0.97 | -7.03 to 5.08 | 0.7528 |
|  | Current drinking | -0.17 | -6.23 to 5.90 | 0.9570 |
| **L Total Hip T-score** |  |  |  |  |
|  | Cluster 2 vs 1 | 0.55 | 0.14 to 0.95 | 0.0090 |
|  | Cluster 3 vs 1 | 0.09 | -0.47 to 0.65 | 0.7421 |
|  | Cluster 4 vs 1 | 1.12 | 0.63 to 1.60 | <0.001 |
|  | Age (per year) | -0.01 | -0.03 to -0.00 | 0.0072 |
|  | Male sex | -0.24 | -0.77 to 0.28 | 0.3640 |
|  | Current smoking | -0.29 | -0.67 to 0.08 | 0.1309 |
|  | Current drinking | 0.03 | -0.34 to 0.40 | 0.8768 |
| **HDL-C, mmol/L** |  |  |  |  |
|  | Cluster 2 vs 1 | -0.11 | -0.22 to -0.00 | 0.0469 |
|  | Cluster 3 vs 1 | -0.24 | -0.39 to -0.09 | 0.0021 |
|  | Cluster 4 vs 1 | -0.23 | -0.37 to -0.09 | 0.0013 |
|  | Age (per year) | 0.00 | -0.00 to 0.00 | 0.5609 |
|  | Male sex | -0.31 | -0.45 to -0.16 | <0.001 |
|  | Current smoking | -0.05 | -0.15 to 0.05 | 0.3439 |
|  | Current drinking | 0.03 | -0.06 to 0.13 | 0.5086 |
| **TG, mmol/L** |  |  |  |  |
|  | Cluster 2 vs 1 | 0.94 | 0.03 to 1.85 | 0.0445 |
|  | Cluster 3 vs 1 | 0.59 | -0.66 to 1.84 | 0.3587 |
|  | Cluster 4 vs 1 | 0.93 | -0.22 to 2.08 | 0.1138 |
|  | Age (per year) | -0.01 | -0.03 to 0.01 | 0.3952 |
|  | Male sex | 0.57 | -0.64 to 1.79 | 0.3575 |
|  | Current smoking | -0.31 | -1.13 to 0.52 | 0.4659 |
|  | Current drinking | 0.77 | -0.06 to 1.60 | 0.0697 |
| **HOMA-IR** |  |  |  |  |
|  | Cluster 2 vs 1 | -0.06 | -1.75 to 1.63 | 0.9435 |
|  | Cluster 3 vs 1 | -1.41 | -3.72 to 0.90 | 0.2334 |
|  | Cluster 4 vs 1 | 2.11 | 0.00 to 4.22 | 0.0518 |
|  | Age (per year) | 0.02 | -0.03 to 0.06 | 0.4549 |
|  | Male sex | -0.88 | -3.08 to 1.33 | 0.4368 |
|  | Current smoking | -0.83 | -2.37 to 0.72 | 0.2957 |
|  | Current drinking | 0.26 | -1.29 to 1.80 | 0.7461 |
| All models adjusted for cluster membership, age, sex, smoking, and alcohol consumption. Reference: Cluster 1, Female sex, Non-smoker, Non-drinker. | | | | |
