# Supplementary material for: Body Composition Phenotypes and Their Metabolic Associations in Treatment‐Naïve Patients With Newly Diagnosed Type 2 Diabetes: A DXA‐Based Cluster Analysis
Source: Endocrinol Diabetes Metab. 2026 Oct 19;9(6):e70366. doi: 10.1002/edm2.70366 (PMC13680683; doi:10.1002/edm2.70366)
Supplement: Supplementary file 4 [file EDM2-9-e70366-s004.docx]

# Supplemental Table S6. Distribution of chronic complications and comorbidities across body composition clusters (corrected).

| **Condition** | **Cluster** | **N** | **With Condition** | **Without Condition** | **Unknown** | **Prevalence (%)** |
| --- | --- | --- | --- | --- | --- | --- |
| Osteoporosis | Cluster 1 | 43 | 19 | 22 | 2 | 46.3% |
|  | Cluster 2 | 72 | 20 | 47 | 5 | 29.9% |
|  | Cluster 3 | 50 | 20 | 23 | 7 | 46.5% |
|  | Cluster 4 | 24 | 2 | 21 | 1 | 8.7% |
|  | Overall, P = 0.0046 | 186 | 61 | 113 | 12 | 35.1% |
|  |  |  |  |  |  |  |
| MASLD (NAFLD) | Cluster 1 | 43 | 12 | 25 | 6 | 32.4% |
|  | Cluster 2 | 72 | 51 | 12 | 9 | 81.0% |
|  | Cluster 3 | 50 | 22 | 16 | 12 | 57.9% |
|  | Cluster 4 | 24 | 15 | 4 | 5 | 78.9% |
|  | Overall, P = <0.001 | 186 | 100 | 57 | 29 | 63.7% |
|  |  |  |  |  |  |  |
| Hyperuricemia | Cluster 1 | 43 | 3 | 39 | 1 | 7.1% |
|  | Cluster 2 | 72 | 15 | 56 | 1 | 21.1% |
|  | Cluster 3 | 50 | 9 | 40 | 1 | 18.4% |
|  | Cluster 4 | 24 | 9 | 14 | 1 | 39.1% |
|  | Overall, P = 0.0176 | 186 | 36 | 150 | 0 | 19.4% |
|  |  |  |  |  |  |  |
| Limb Arteriosclerosis | Cluster 1 | 43 | 25 | 8 | 10 | 75.8% |
|  | Cluster 2 | 72 | 31 | 25 | 16 | 55.4% |
|  | Cluster 3 | 50 | 15 | 6 | 29 | 71.4% |
|  | Cluster 4 | 24 | 7 | 10 | 7 | 41.2% |
|  | Overall, P = 0.0536 | 186 | 78 | 49 | 59 | 61.4% |
|  |  |  |  |  |  |  |
| Diabetic Kidney Disease | Cluster 1 | 43 | 8 | 34 | 1 | 19.0% |
|  | Cluster 2 | 72 | 15 | 56 | 1 | 21.1% |
|  | Cluster 3 | 50 | 15 | 34 | 1 | 30.6% |
|  | Cluster 4 | 24 | 7 | 16 | 1 | 30.4% |
|  | Overall, P = 0.4754 | 186 | 45 | 140 | 1 | 24.3% |
|  |  |  |  |  |  |  |
| Diabetic Retinopathy | Cluster 1 | 43 | 2 | 29 | 12 | 6.5% |
|  | Cluster 2 | 72 | 4 | 45 | 23 | 8.2% |
|  | Cluster 3 | 50 | 1 | 14 | 35 | 6.7% |
|  | Cluster 4 | 24 | 1 | 9 | 14 | 10.0% |
|  | Overall, P = 1.0000 | 186 | 8 | 97 | 81 | 7.6% |
|  |  |  |  |  |  |  |
| Carotid Plaque | Cluster 1 | 43 | 21 | 9 | 13 | 70.0% |
|  | Cluster 2 | 72 | 35 | 13 | 24 | 72.9% |
|  | Cluster 3 | 50 | 11 | 4 | 35 | 73.3% |
|  | Cluster 4 | 24 | 10 | 6 | 8 | 62.5% |
|  | Overall, P = 0.8779 | 186 | 77 | 32 | 77 | 70.6% |
|  |  |  |  |  |  |  |
| Hypertension | Cluster 1 | 43 | 16 | 21 | 6 | 43.2% |
|  | Cluster 2 | 72 | 30 | 35 | 7 | 46.2% |
|  | Cluster 3 | 50 | 19 | 20 | 11 | 48.7% |
|  | Cluster 4 | 24 | 10 | 9 | 5 | 52.6% |
|  | Overall, P = 0.9068 | 186 | 75 | 85 | 26 | 46.9% |
|  |  |  |  |  |  |  |
| Coronary Artery Disease | Cluster 1 | 43 | 5 | 32 | 6 | 13.5% |
|  | Cluster 2 | 72 | 9 | 54 | 9 | 14.3% |
|  | Cluster 3 | 50 | 2 | 38 | 10 | 5.0% |
|  | Cluster 4 | 24 | 1 | 18 | 5 | 5.3% |
|  | Overall, P = 0.4134 | 186 | 17 | 142 | 27 | 10.7% |
|  |  |  |  |  |  |  |
| Dyslipidemia | Cluster 1 | 43 | 19 | 18 | 6 | 51.4% |
|  | Cluster 2 | 72 | 40 | 25 | 7 | 61.5% |
|  | Cluster 3 | 50 | 22 | 20 | 8 | 52.4% |
|  | Cluster 4 | 24 | 14 | 7 | 3 | 66.7% |
|  | Overall, P = 0.5495 | 186 | 95 | 70 | 21 | 57.6% |
|  |  |  |  |  |  |  |
| Prevalence = percentage of patients with known status (% within column). P-values from Fisher exact test (simulated, 10,000 replicates) on known cases only. DR analysis limited by 43.5% missing data (8 cases among 105 with known status). | | | | | | |
