# Supplementary material for: Body Composition Phenotypes and Their Metabolic Associations in Treatment‐Naïve Patients With Newly Diagnosed Type 2 Diabetes: A DXA‐Based Cluster Analysis
Source: Endocrinol Diabetes Metab. 2026 Oct 19;9(6):e70366. doi: 10.1002/edm2.70366 (PMC13680683; doi:10.1002/edm2.70366)
Supplement: Supplementary file 5 [file EDM2-9-e70366-s005.docx]

# Supplemental Table S8. Missing data report for variables used in complication and regression analyses (N = 186).

| **Variable** | **N Missing** | **% Missing** | **N Available** | **Likely Reason** |
| --- | --- | --- | --- | --- |
| Diabetic Retinopathy | 81 | 43.5% | 105 | Fundus examination not performed |
| Carotid Plaque | 77 | 41.4% | 109 | Carotid ultrasound not performed |
| Limb Arteriosclerosis | 59 | 31.7% | 127 | Lower extremity ultrasound not performed |
| MASLD (NAFLD) | 29 | 15.6% | 157 | Liver ultrasound not performed |
| Coronary Artery Disease | 27 | 14.5% | 159 | CTCA/coronary angiography not performed |
| Hypertension | 26 | 14.0% | 160 | Not recorded |
| Dyslipidemia | 21 | 11.3% | 165 | Not recorded |
| Osteoporosis | 12 | 6.5% | 174 | DXA not performed |
| Hyperuricemia | 0 | 0.0% | 186 | Not measured |
| Diabetic Kidney Disease | 1 | 0.5% | 185 | UACR/eGFR not recorded |
| Smoking Status | 22 | 11.8% | 164 | Self-report not recorded |
| Drinking Status | 21 | 11.3% | 165 | Self-report not recorded |
| Age | 12 | 6.5% | 174 | Not recorded |
| Sex | 1 | 0.5% | 185 | Not recorded |
| Multiple imputation (MICE, 20 datasets) used to address missing data in regression models. Missing at random (MAR) assumption cannot be verified. Variables with >30% missing (DR, carotid plaque, LEAD) should be interpreted with caution. | | | | |
