# Supplementary material for: Body Composition Phenotypes and Their Metabolic Associations in Treatment‐Naïve Patients With Newly Diagnosed Type 2 Diabetes: A DXA‐Based Cluster Analysis
Source: Endocrinol Diabetes Metab. 2026 Oct 19;9(6):e70366. doi: 10.1002/edm2.70366 (PMC13680683; doi:10.1002/edm2.70366)
Supplement: Supplementary file 6 [file EDM2-9-e70366-s006.docx]

# Supplemental Table S11. Sex-stratified clustering: within-sex body-composition phenotypes, k-selection, and metabolic associations.

## A. k-selection by sex (silhouette width and Calinski-Harabasz index)

| **Sex** | **k** | **Silhouette** | **CH index** | **Cluster sizes** |
| --- | --- | --- | --- | --- |
| Female | 2 | 0.327 | 43.1 | 49, 22 |
| Female | 3 | 0.250 | 37.2 | 18, 37, 16 |
| Female | 4 | 0.210 | 30.9 | 17, 17, 5, 32 |
| Male | 2 | 0.325 | 67.3 | 76, 38 |
| Male | 3 | 0.257 | 58.6 | 41, 14, 59 |
| Male | 4 | 0.183 | 48.2 | 25, 47, 13, 29 |

## B. Within-sex cluster phenotypes (male k=3, female k=2)

| **Phenotype** | **n** | **Total fat (g)** | **Total lean (g)** | **A/G ratio** | **VAT (g)** | **ALMI** | **FMI** |
| --- | --- | --- | --- | --- | --- | --- | --- |
| Female lean/low-adiposity | 49 | 25,169 | 33,820 | 1.11 | 638 | 5.8 | 9.9 |
| Female high-adiposity | 22 | 40,295 | 42,084 | 1.16 | 1,058 | 7.3 | 15.2 |
| Male low-fat/lean | 41 | 21,792 | 43,392 | 1.26 | 616 | 6.6 | 7.4 |
| Male intermediate (highest A/G) | 59 | 29,999 | 51,784 | 1.34 | 906 | 8.0 | 9.8 |
| Male central-obesity/high-muscle | 14 | 45,851 | 60,311 | 1.28 | 993 | 9.4 | 14.6 |

Methods: Clustering was performed separately within males (n=114) and females (n=71) using the same 50 DXA-derived variables (LArmFatMassg to AppenLeanBMCHt2kgm2), Z-score standardized within each sex. The number of clusters (male k=3, female k=2) was chosen by the silhouette and elbow methods; silhouette widths for k=2-4 are shown in Panel A. Male clustering distinguished a low-fat/lean phenotype, an intermediate phenotype with the highest A/G ratio (central adiposity), and a central-obesity/high-muscle phenotype. Female clustering distinguished a lean/low-adiposity phenotype from a high-adiposity phenotype.
