# Supplementary material for: Body Composition Phenotypes and Their Metabolic Associations in Treatment‐Naïve Patients With Newly Diagnosed Type 2 Diabetes: A DXA‐Based Cluster Analysis
Source: Endocrinol Diabetes Metab. 2026 Oct 19;9(6):e70366. doi: 10.1002/edm2.70366 (PMC13680683; doi:10.1002/edm2.70366)
Supplement: Supplementary file 7 [file EDM2-9-e70366-s007.docx]

# Supplemental Table S4. Variance decomposition: unique contributions of cluster membership and sex to DXA-derived body composition variables.

| **Variable** | **R2(Cluster)** | **R2(Sex)** | **R2(Cluster+Sex)** | **dR2(Cluster\|Sex)** | **dR2(Sex\|Cluster)** |
| --- | --- | --- | --- | --- | --- |
| Total Fat Mass(g) | 0.7459 | 0.0023 | 0.7461 | 0.7438 | 0.0003 |
| Android Fat Mass(g) | 0.6988 | 0.0102 | 0.7019 | 0.6917 | 0.0031 |
| Limb Fat Mass(g) | 0.7027 | 0.0243 | 0.7036 | 0.6793 | 0.0009 |
| Trunk Fat Mass(g) | 0.6391 | 0.0041 | 0.6427 | 0.6386 | 0.0036 |
| L Leg Fat Mass(g) | 0.6595 | 0.0284 | 0.6599 | 0.6315 | 0.0004 |
| FMI(kg/m2) | 0.7044 | 0.1001 | 0.7218 | 0.6217 | 0.0174 |
| R Arm Fat Mass(g) | 0.6288 | 0.0376 | 0.6340 | 0.5964 | 0.0052 |
| L Arm Fat Mass(g) | 0.6387 | 0.0552 | 0.6480 | 0.5928 | 0.0093 |
| Total Total Mass(g) | 0.7022 | 0.1511 | 0.7419 | 0.5907 | 0.0396 |
| R Leg Fat Mass(g) | 0.6198 | 0.0307 | 0.6201 | 0.5894 | 0.0003 |
| L Arm Total Mass(g) | 0.6331 | 0.0779 | 0.6483 | 0.5704 | 0.0153 |
| R Arm Total Mass(g) | 0.6220 | 0.0834 | 0.6465 | 0.5631 | 0.0245 |
| L Leg Total Mass(g) | 0.6423 | 0.1179 | 0.6779 | 0.5601 | 0.0356 |
| R Leg Total Mass(g) | 0.6274 | 0.1235 | 0.6687 | 0.5452 | 0.0413 |
| Gynoid Fat Mass(g) | 0.5254 | 0.0752 | 0.5254 | 0.4502 | 0.0000 |
| Trunk Total Mass(g) | 0.5486 | 0.1451 | 0.5875 | 0.4424 | 0.0389 |
| Trunk Fat(%) | 0.5992 | 0.1761 | 0.6136 | 0.4375 | 0.0144 |
| Est. VAT Area(cm2) | 0.3995 | 0.0067 | 0.4028 | 0.3961 | 0.0032 |
| Est. VAT Mass(g) | 0.3991 | 0.0067 | 0.4024 | 0.3957 | 0.0032 |
| ALMI(kg/m2) | 0.6158 | 0.2743 | 0.6549 | 0.3806 | 0.0392 |
| Lean+BMC Index, kg/m2 | 0.5917 | 0.2581 | 0.6235 | 0.3655 | 0.0319 |
| Total Body Fat(%)(derived) | 0.6916 | 0.3815 | 0.7437 | 0.3622 | 0.0521 |
| Total Body Fat(%)(total) | 0.6996 | 0.3896 | 0.7508 | 0.3612 | 0.0512 |
| L Leg Lean Mass(g) | 0.6206 | 0.3983 | 0.7025 | 0.3042 | 0.0818 |
| R Leg Lean Mass(g) | 0.6111 | 0.4093 | 0.7052 | 0.2959 | 0.0942 |
| Limb Lean Mass(g) | 0.6298 | 0.4519 | 0.7247 | 0.2728 | 0.0948 |
| L Arm Fat(%) | 0.6965 | 0.5364 | 0.7939 | 0.2575 | 0.0974 |
| R Arm Fat(%) | 0.6672 | 0.5096 | 0.7644 | 0.2548 | 0.0972 |
| Total Lean Mass(g) | 0.6330 | 0.4858 | 0.7371 | 0.2512 | 0.1041 |
| L Leg Fat(%) | 0.6384 | 0.4624 | 0.6956 | 0.2332 | 0.0572 |
| R Leg Fat(%) | 0.6201 | 0.4684 | 0.6837 | 0.2153 | 0.0637 |
| L Arm Lean Mass(g) | 0.5851 | 0.4973 | 0.6809 | 0.1835 | 0.0957 |
| R Arm Lean Mass(g) | 0.5834 | 0.5275 | 0.7065 | 0.1789 | 0.1230 |
| Trunk Lean Mass(g) | 0.4466 | 0.3745 | 0.5291 | 0.1547 | 0.0825 |
| Android/Gynoid Ratio | 0.3074 | 0.3290 | 0.3758 | 0.0467 | 0.0684 |
| dR2(Cluster\|Sex) = R2(Cluster+Sex) - R2(Sex): unique variance explained by cluster beyond sex. dR2(Sex\|Cluster) = R2(Cluster+Sex) - R2(Cluster): unique variance explained by sex beyond cluster. Variables sorted by descending dR2(Cluster\|Sex). | | | | | |
