# Supplementary material for: Body Composition Phenotypes and Their Metabolic Associations in Treatment‐Naïve Patients With Newly Diagnosed Type 2 Diabetes: A DXA‐Based Cluster Analysis
Source: Endocrinol Diabetes Metab. 2026 Oct 19;9(6):e70366. doi: 10.1002/edm2.70366 (PMC13680683; doi:10.1002/edm2.70366)
Supplement: Supplementary file 8 [file EDM2-9-e70366-s009.docx]

# Supplemental Table S10. Incremental clinical utility of the clustering framework beyond conventional indices (BMI, BMI+WC, and BMI+VAT): nested model comparison.

| **Outcome** | **Type** | **Δ (BMI)** | **P (BMI)** | **Δ (BMI+WC)** | **P (BMI+WC)** | **Δ (BMI+VAT)** | **P (BMI+VAT)** |
| --- | --- | --- | --- | --- | --- | --- | --- |
| Uric acid | Continuous | 0.038 | 0.092 | 0.034 | 0.144 | 0.030 | 0.154 |
| TG | Continuous | 0.012 | 0.621 | 0.007 | 0.782 | 0.009 | 0.722 |
| HDL-C | Continuous | 0.024 | 0.236 | 0.017 | 0.405 | 0.023 | 0.241 |
| ALT | Continuous | 0.006 | 0.840 | 0.007 | 0.825 | 0.003 | 0.940 |
| BUN | Continuous | 0.050 | 0.067 | 0.060 | 0.043 | 0.073 | 0.014 |
| Hip T-score | Continuous | 0.014 | 0.441 | 0.009 | 0.641 | 0.012 | 0.517 |
| γ-GGT | Continuous | 0.012 | 0.609 | 0.013 | 0.601 | 0.008 | 0.747 |
| HbA1c | Continuous | 0.010 | 0.741 | 0.015 | 0.613 | 0.009 | 0.766 |
| eGFR | Continuous | 0.011 | 0.348 | 0.009 | 0.483 | 0.014 | 0.243 |
| MASLD | Binary | 0.076 | 0.003 | 0.068 | 0.008 | 0.064 | 0.009 |
| Osteoporosis | Binary | 0.008 | 0.657 | 0.010 | 0.599 | 0.007 | 0.706 |
| Hyperuricemia | Binary | 0.017 | 0.445 | 0.014 | 0.564 | 0.017 | 0.457 |

All models adjust for sex, age, smoking, and drinking. Three conventional-control models were compared: (1) BMI, (2) BMI + waist circumference (WC), and (3) BMI + visceral adipose tissue area (VAT). Continuous outcomes use linear regression (ΔR² and F-test P); binary outcomes use logistic regression (Δ McFadden pseudo-R² and likelihood-ratio test P). P < 0.05 indicates that cluster significantly improves prediction beyond the conventional indices.
