# Supplementary material for: Body Composition Phenotypes and Their Metabolic Associations in Treatment‐Naïve Patients With Newly Diagnosed Type 2 Diabetes: A DXA‐Based Cluster Analysis
Source: Endocrinol Diabetes Metab. 2026 Oct 19;9(6):e70366. doi: 10.1002/edm2.70366 (PMC13680683; doi:10.1002/edm2.70366)
Supplement: Supplementary file 9 [file EDM2-9-e70366-s010.docx]

# Supplemental Table S3. Nested model comparison: variance explained by cluster alone (Model 1) vs cluster + sex (Model 2) for key metabolic outcomes.

| **Variable** | **Cluster P** | **R2 (Model 1)** | **Cluster+Sex P** | **R2 (Model 2)** | **dR2** | **Nested F P** |
| --- | --- | --- | --- | --- | --- | --- |
| ALT(U/L) | 2.99e-03 | 0.0913 | 2.98e-03 | 0.0987 | 0.0073 | 0.2817 |
| AST(U/L) | 1.77e-02 | 0.0662 | 1.76e-02 | 0.0735 | 0.0073 | 0.2855 |
| γ-GGT(U/L) | 5.01e-03 | 0.0844 | 5.00e-03 | 0.0913 | 0.0069 | 0.2977 |
| UA(μmol/L) | 1.94e-05 | 0.1393 | 1.70e-05 | 0.1551 | 0.0158 | 0.0834 |
| BUN(mmol/L) | 5.11e-05 | 0.1596 | 5.14e-05 | 0.1662 | 0.0066 | 0.3153 |
| Cr(μmol/L) | 1.86e-02 | 0.0590 | 1.14e-02 | 0.1580 | 0.0990 | 0.0000 |
| L Total Hip T-score | 5.34e-06 | 0.1524 | 5.78e-06 | 0.1524 | 0.0000 | 0.9646 |
| L Total Hip Z-score | 3.11e-05 | 0.1326 | 2.43e-05 | 0.1580 | 0.0254 | 0.0275 |
| HDL-C(mmol/L) | 2.91e-06 | 0.1597 | 5.10e-07 | 0.2674 | 0.1076 | 0.0000 |
| TG(mmol/L) | 2.18e-03 | 0.0855 | 2.13e-03 | 0.0947 | 0.0092 | 0.2019 |
| HOMA-IR | 3.95e-02 | 0.0529 | 3.97e-02 | 0.0583 | 0.0055 | 0.3497 |
| HOMA-β | 4.79e-01 | 0.0151 | 4.77e-01 | 0.0243 | 0.0092 | 0.2176 |
| Model 1: outcome ~ cluster. Model 2: outcome ~ cluster + sex. dR2 = R2(Model 2) - R2(Model 1). Nested F-test P assesses significance of incremental contribution of sex. | | | | | | |
