# Supplementary material for: Body Composition Phenotypes and Their Metabolic Associations in Treatment‐Naïve Patients With Newly Diagnosed Type 2 Diabetes: A DXA‐Based Cluster Analysis
Source: Endocrinol Diabetes Metab. 2026 Oct 19;9(6):e70366. doi: 10.1002/edm2.70366 (PMC13680683; doi:10.1002/edm2.70366)
Supplement: Supplementary file 10 [file EDM2-9-e70366-s011.docx]

# Supplemental Table S1. Baseline characteristics, body composition, and metabolic profiles of treatment-naive T2DM patients stratified by body composition cluster (N = 185).

| **Characteristic** | **Overall (N=185)** | **Cluster 1 (N=42)** | **Cluster 2 (N=71)** | **Cluster 3 (N=49)** | **Cluster 4 (N=23)** | **P-value (raw)** | **P-value (adjusted)** |
| --- | --- | --- | --- | --- | --- | --- | --- |
| **Demographics and Clinical Characteristics** |  |  |  |  |  |  |  |
| Female | 71 (38%) | 14 (33%) | 1 (1%) | 48 (98%) | 8 (35%) | <0.001 | <0.001 |
| Male | 114 (62%) | 28 (67%) | 70 (99%) | 1 (2%) | 15 (65%) | <0.001 | <0.001 |
| Age, years | 52.0 (38.0-62.0) | 60.0 (49.0-67.0) | 47.0 (36.5-56.5) | 56.0 (39.0-63.0) | 41.0 (34.0-55.0) | 0.001 | 0.006 |
| Height, m | 1.7 (1.6-1.8) | 1.7 (1.6-1.7) | 1.8 (1.7-1.8) | 1.6 (1.6-1.6) | 1.8 (1.6-1.8) | 0.000 | 0.000 |
| Weight, kg | 76.0 (65.0-85.6) | 62.2 (56.5-67.5) | 82.0 (76.5-88.0) | 68.0 (62.4-75.2) | 106.0 (97.0-110.0) | 0.000 | 0.000 |
| BMI, kg/m2 | 25.9 (23.6-29.8) | 21.8 (20.3-23.1) | 26.9 (25.1-29.4) | 26.3 (24.4-27.5) | 35.1 (32.3-37.6) | 0.000 | 0.000 |
| Waist circumference, cm | 94.0 (87.0-100.0) | 83.0 (77.8-87.0) | 96.0 (92.0-100.0) | 92.0 (87.5-96.0) | 115.0 (107.0-121.0) | 0.000 | 0.000 |
| SBP, mmHg | 129.0 (121.0-139.0) | 128.0 (122.5-137.0) | 129.5 (122.2-139.8) | 129.0 (118.0-140.0) | 132.5 (123.8-139.2) | 0.773 | 1.000 |
| DBP, mmHg | 82.0 (76.0-89.0) | 81.0 (74.5-87.0) | 83.5 (77.2-90.8) | 79.0 (75.0-87.5) | 82.0 (79.8-88.2) | 0.380 | 1.000 |
| Non-smoker | 102 (55%) | 21 (50%) | 31 (44%) | 36 (73%) | 14 (61%) | <0.001 | <0.001 |
| Current smoker | 62 (34%) | 15 (36%) | 35 (49%) | 4 (8%) | 8 (35%) | <0.001 | <0.001 |
| Non-drinker | 118 (64%) | 25 (60%) | 41 (58%) | 36 (73%) | 16 (70%) | <0.001 | <0.001 |
| Current drinker | 47 (25%) | 11 (26%) | 25 (35%) | 5 (10%) | 6 (26%) | <0.001 | <0.001 |
| **Body Composition (DXA-Derived, Cluster-Defining Variables - Descriptive Only)** |  |  |  |  |  |  |  |
| L Arm Fat Mass, g | 1702.4 (1374.5-2162.2) | 1107.8 (938.9-1321.8) | 1656.3 (1485.1-1902.8) | 2046.3 (1661.9-2476.8) | 3312.3 (2763.5-3541.7) | - | - |
| L Arm Lean Mass, g | 2249.2 (1826.7-2776.3) | 2092.9 (1802.2-2208.0) | 2763.6 (2418.8-3022.9) | 1726.8 (1506.5-1854.9) | 2802.7 (2366.2-3305.8) | - | - |
| L Arm Total Mass, g | 4216.5 (3571.4-4933.8) | 3389.2 (3040.4-3667.8) | 4556.6 (4212.4-5070.1) | 3914.3 (3308.9-4380.2) | 6019.5 (5745.4-6919.1) | - | - |
| L Arm Fat, % | 40.5 (35.1-51.1) | 35.0 (29.4-38.5) | 36.4 (33.2-39.1) | 53.7 (50.9-56.6) | 49.7 (45.1-57.9) | - | - |
| R Arm Fat Mass, g | 1754.7 (1414.4-2319.9) | 1156.9 (1044.9-1328.5) | 1738.8 (1561.1-1969.7) | 2147.3 (1721.8-2637.6) | 3430.3 (2909.9-3701.7) | - | - |
| R Arm Lean Mass, g | 2496.2 (2052.1-3049.3) | 2303.2 (1864.3-2493.3) | 2981.2 (2724.2-3313.1) | 1949.5 (1672.8-2121.9) | 3088.7 (2681.8-3631.1) | - | - |
| R Arm Total Mass, g | 4595.3 (3867.0-5381.9) | 3704.4 (3231.8-3921.2) | 4963.3 (4523.2-5420.5) | 4212.1 (3633.3-4867.1) | 6660.2 (6074.6-7326.9) | - | - |
| R Arm Fat, % | 38.9 (33.6-49.7) | 32.5 (28.7-38.6) | 35.5 (32.8-37.5) | 52.7 (48.3-56.0) | 49.7 (44.0-58.0) | - | - |
| Trunk Fat Mass, g | 14488.7 (12253.2-17661.5) | 10895.0 (8816.9-12234.0) | 15071.2 (13724.8-16946.6) | 15408.6 (12378.5-16765.7) | 23281.4 (20395.7-25051.8) | - | - |
| Trunk Lean Mass, g | 20315.1 (17195.9-23352.9) | 18466.6 (16642.1-21401.1) | 22329.2 (20212.2-25037.9) | 16728.9 (14783.7-17771.9) | 24621.8 (22520.8-26012.7) | - | - |
| Trunk Total Mass, g | 35663.6 (31259.4-40831.1) | 30769.4 (25591.7-34212.5) | 38347.3 (35277.6-41988.6) | 32253.3 (28464.7-35922.7) | 47575.4 (43964.3-51990.7) | - | - |
| Trunk Fat, % | 40.5 (37.1-45.7) | 35.5 (32.5-37.9) | 40.0 (37.4-41.5) | 46.1 (43.5-49.2) | 48.6 (46.5-50.4) | - | - |
| L Leg Fat Mass, g | 4151.3 (3349.8-5015.7) | 2885.8 (2435.8-3157.7) | 4129.7 (3734.8-4695.9) | 4810.5 (4396.4-5247.3) | 7024.9 (6029.1-7786.4) | - | - |
| L Leg Lean Mass, g | 7774.5 (6467.3-9105.7) | 6619.1 (5629.3-7157.0) | 9035.1 (8531.6-9786.8) | 6421.5 (5628.6-6828.2) | 10276.8 (8318.0-11340.0) | - | - |
| L Leg Total Mass, g | 12464.8 (10678.8-14216.2) | 9749.5 (8973.3-11003.6) | 13888.1 (12664.0-14628.5) | 11485.6 (10380.8-12519.5) | 17786.7 (14964.2-19573.1) | - | - |
| L Leg Fat, % | 33.4 (29.0-39.2) | 29.4 (25.3-33.3) | 30.0 (27.9-32.4) | 42.4 (38.7-44.6) | 40.2 (37.0-44.1) | - | - |
| R Leg Fat Mass, g | 4168.9 (3255.0-5079.6) | 2787.5 (2439.8-3199.4) | 4040.9 (3602.2-4694.7) | 4809.7 (4168.9-5376.7) | 6681.1 (5974.3-8070.5) | - | - |
| R Leg Lean Mass, g | 7794.5 (6515.4-9176.7) | 6694.4 (5393.4-7305.3) | 9163.5 (8646.0-9663.2) | 6439.0 (5764.6-6792.2) | 10257.8 (8251.5-11312.2) | - | - |
| R Leg Total Mass, g | 12516.7 (10589.3-14195.1) | 9821.9 (8755.2-10900.1) | 13871.7 (12823.2-14586.5) | 11553.8 (10385.4-12773.5) | 17806.8 (14680.9-19622.0) | - | - |
| R Leg Fat, % | 32.9 (28.8-39.2) | 29.6 (25.3-33.0) | 29.8 (27.4-32.2) | 42.1 (38.0-44.8) | 39.4 (36.3-43.5) | - | - |
| Total Fat Mass, g | 27665.1 (24112.1-33457.6) | 19812.2 (17481.9-22146.3) | 28526.5 (26424.9-30947.5) | 29790.9 (26188.4-33457.6) | 44086.2 (41443.4-49418.1) | - | - |
| Total Lean Mass, g | 44091.9 (37787.1-51185.5) | 40138.1 (36168.8-43357.8) | 49974.7 (46808.8-54020.5) | 35371.8 (32370.2-38697.9) | 54865.8 (50061.2-58435.7) | - | - |
| Total Total Mass, g | 75262.2 (65396.4-84984.0) | 61255.4 (55549.8-66280.6) | 80884.2 (76311.1-87387.6) | 67184.0 (61502.1-73321.4) | 104565.7 (96267.7-108707.7) | - | - |
| Total Body Fat, % | 36.9 (33.6-43.1) | 33.0 (30.3-34.9) | 35.1 (33.5-36.7) | 44.3 (41.7-46.4) | 45.6 (41.8-47.3) | - | - |
| Limb Fat Mass, g | 13288.5 (10967.4-16350.6) | 9292.0 (8120.3-10552.5) | 13193.9 (12304.0-14970.5) | 14932.8 (12652.2-17167.7) | 21845.7 (19870.1-25267.5) | - | - |
| Limb Lean Mass, g | 23923.5 (20488.1-27729.8) | 21162.3 (18148.4-22329.5) | 27540.8 (26381.4-29372.7) | 19433.7 (17358.2-20700.8) | 31219.0 (24821.0-34020.1) | - | - |
| Android Fat Mass, g | 2567.3 (2077.5-3184.2) | 1782.6 (1479.8-2048.0) | 2730.6 (2404.0-3134.3) | 2594.5 (2168.2-2753.3) | 4481.5 (3991.6-4778.6) | - | - |
| Gynoid Fat Mass, g | 19368.4 (18007.6-21681.7) | 16919.0 (15637.6-18109.7) | 19005.5 (18211.7-19731.3) | 21727.1 (20457.0-23405.3) | 23087.8 (22316.7-23518.7) | - | - |
| Total Body Fat, % | 37.2 (33.7-43.2) | 33.0 (30.3-34.9) | 35.1 (33.5-36.8) | 44.3 (41.9-46.7) | 45.6 (41.8-47.3) | - | - |
| FMI, kg/m2 | 9.8 (8.2-11.9) | 7.0 (6.4-7.5) | 9.3 (8.6-10.4) | 11.6 (10.2-13.1) | 15.6 (13.4-17.6) | - | - |
| Android/Gynoid Ratio | 1.2 (1.1-1.3) | 1.2 (1.1-1.3) | 1.3 (1.3-1.4) | 1.1 (1.1-1.2) | 1.2 (1.2-1.4) | - | - |
| Est. VAT Mass, g | 759.0 (618.0-936.0) | 554.5 (505.2-678.8) | 879.0 (704.0-970.5) | 753.0 (664.0-841.0) | 1106.0 (860.5-1399.0) | - | - |
| Est. VAT Area, cm2 | 157.0 (128.0-194.0) | 115.0 (104.5-140.5) | 182.0 (146.0-201.5) | 156.0 (138.0-174.0) | 229.0 (178.5-290.0) | - | - |
| Lean+BMC Index, kg/m2 | 15.3 (13.8-17.0) | 14.1 (12.6-14.9) | 16.5 (15.5-17.6) | 13.8 (12.9-14.4) | 18.0 (17.4-19.4) | - | - |
| ALMI, kg/m2 | 7.2 (6.2-8.0) | 6.1 (5.4-6.6) | 7.8 (7.5-8.3) | 6.3 (5.5-6.7) | 8.6 (8.0-9.5) | - | - |
| **Glycemic Parameters and Pancreatic Function** |  |  |  |  |  |  |  |
| HbA1c, % | 9.4 (7.5-11.3) | 9.2 (7.6-10.6) | 9.6 (7.9-11.5) | 8.2 (6.5-11.2) | 9.9 (9.0-10.8) | 0.381 | 1.000 |
| FPG, mmol/L | 7.3 (6.1-9.3) | 7.6 (6.3-10.8) | 7.6 (6.0-9.1) | 6.8 (6.0-8.3) | 7.5 (6.8-10.1) | 0.292 | 1.000 |
| Glu 0.5h | 11.5 (9.2-13.5) | 11.6 (9.3-13.5) | 12.3 (9.4-13.9) | 11.1 (8.9-12.5) | 11.0 (9.0-13.3) | 0.579 | 1.000 |
| Glu 1h | 14.6 (12.2-17.2) | 14.8 (12.2-16.4) | 15.3 (12.5-17.7) | 13.5 (11.9-16.9) | 15.4 (11.6-17.2) | 0.692 | 1.000 |
| Glu 2h | 15.9 (12.8-19.9) | 17.1 (14.1-20.0) | 16.4 (12.6-19.8) | 15.4 (12.3-20.0) | 15.8 (14.5-17.7) | 0.908 | 1.000 |
| Glu 3h | 14.1 (9.7-17.0) | 14.5 (9.7-17.7) | 14.0 (10.3-16.2) | 14.9 (9.4-18.0) | 12.6 (11.7-15.6) | 0.837 | 1.000 |
| CP 0h, ng/mL | 2.1 (1.3-3.0) | 1.3 (0.9-1.9) | 2.2 (1.6-3.1) | 2.2 (1.4-3.0) | 2.9 (2.3-3.6) | 0.000 | 0.000 |
| CP 0.5h | 3.1 (2.0-4.8) | 2.2 (1.2-3.3) | 3.7 (2.0-4.9) | 3.1 (2.1-5.5) | 3.6 (2.8-5.5) | 0.008 | 0.163 |
| CP 1h | 4.4 (2.5-7.0) | 3.3 (1.9-5.1) | 4.7 (3.0-6.8) | 4.5 (2.5-8.3) | 5.5 (4.5-8.5) | 0.031 | 0.627 |
| CP 2h | 6.5 (3.8-9.0) | 4.4 (2.8-7.6) | 6.4 (4.4-8.7) | 7.3 (4.1-12.3) | 7.9 (3.8-10.7) | 0.020 | 0.396 |
| CP 3h | 6.4 (4.0-9.0) | 5.5 (3.3-8.3) | 6.4 (4.9-7.9) | 6.8 (3.7-11.2) | 8.4 (5.2-11.1) | 0.304 | 1.000 |
| AUC C-peptide | 16.3 (9.9-21.7) | 13.0 (7.2-18.1) | 16.7 (10.4-21.4) | 15.5 (8.3-24.8) | 19.3 (13.4-28.1) | 0.126 | 1.000 |
| AUC Insulin | 92.7 (47.9-174.5) | 74.7 (47.1-108.1) | 101.1 (47.6-157.1) | 124.0 (48.0-203.9) | 140.4 (70.7-352.1) | 0.090 | 1.000 |
| HOMA-B | 38.7 (32.1-47.3) | 37.1 (29.6-45.4) | 36.9 (32.4-48.0) | 41.3 (35.0-48.9) | 37.7 (30.6-41.7) | 0.247 | 1.000 |
| HOMA-IR | 3.0 (1.8-4.8) | 2.0 (1.0-3.7) | 2.9 (1.8-4.5) | 3.0 (1.9-4.2) | 5.7 (3.7-7.2) | 0.000 | 0.008 |
| **Hepatic Enzyme Profiles** |  |  |  |  |  |  |  |
| ALT, U/L | 25.0 (16.0-38.0) | 17.5 (13.0-24.5) | 28.0 (19.0-40.0) | 22.0 (15.0-33.0) | 45.0 (30.0-75.0) | 0.000 | 0.001 |
| AST, U/L | 19.0 (15.0-30.0) | 16.5 (15.0-22.0) | 20.0 (15.0-28.0) | 18.0 (15.0-31.0) | 28.0 (20.0-53.0) | 0.012 | 0.047 |
| ALP, U/L | 75.0 (62.5-91.0) | 69.5 (62.0-92.8) | 73.5 (60.8-83.2) | 78.0 (65.0-92.2) | 79.0 (68.0-92.0) | 0.146 | 0.584 |
| GGT, U/L | 29.0 (18.0-46.0) | 20.0 (17.0-35.0) | 36.0 (24.0-52.0) | 21.5 (16.8-37.2) | 47.0 (25.0-87.0) | 0.000 | 0.001 |
| **Renal Function Parameters** |  |  |  |  |  |  |  |
| BUN, mmol/L | 5.0 (3.9-5.7) | 5.6 (4.5-6.3) | 5.2 (4.3-6.0) | 4.1 (3.5-5.1) | 4.2 (3.8-5.3) | 0.000 | 0.001 |
| Cr, μmol/L | 63.0 (53.8-72.0) | 62.5 (51.5-76.0) | 67.0 (61.0-76.0) | 54.5 (47.8-61.2) | 63.0 (53.0-73.0) | 0.000 | 0.000 |
| eGFR, mL/min/1.73m2 | 105.0 (95.0-116.0) | 101.5 (91.2-110.8) | 110.0 (97.0-116.0) | 100.0 (94.0-119.0) | 109.0 (101.0-119.0) | 0.127 | 0.762 |
| UA, μmol/L | 334.0 (267.5-395.5) | 281.0 (233.0-343.0) | 369.0 (318.0-409.0) | 303.0 (258.5-349.0) | 384.0 (313.0-429.0) | 0.000 | 0.000 |
| ACR, mg/g | 9.3 (6.6-18.4) | 9.4 (7.7-16.2) | 12.1 (7.0-19.4) | 7.0 (4.6-13.6) | 8.7 (5.4-23.9) | 0.183 | 1.000 |
| UAER | 8.6 (0.0-18.7) | 10.2 (0.0-16.9) | 11.3 (0.0-27.1) | 0.0 (0.0-8.8) | 12.8 (0.0-22.8) | 0.075 | 0.452 |
| **Lipid Profiles** |  |  |  |  |  |  |  |
| TC, mmol/L | 4.7 (4.1-5.4) | 4.6 (3.8-5.4) | 4.8 (4.2-5.5) | 4.8 (4.3-5.4) | 4.4 (3.8-5.0) | 0.304 | 1.000 |
| TG, mmol/L | 1.6 (1.1-2.4) | 1.1 (0.8-1.9) | 1.7 (1.4-2.9) | 1.4 (1.0-2.1) | 1.9 (1.3-2.6) | 0.000 | 0.001 |
| HDL-C, mmol/L | 1.0 (0.8-1.2) | 1.0 (0.9-1.3) | 0.9 (0.8-1.0) | 1.1 (1.0-1.3) | 0.8 (0.8-1.0) | 0.000 | 0.000 |
| LDL-C, mmol/L | 3.2 (2.6-3.7) | 3.1 (2.4-3.9) | 3.2 (2.7-3.9) | 3.2 (2.8-3.6) | 3.2 (2.7-3.4) | 0.717 | 1.000 |
| **Bone Turnover Markers** |  |  |  |  |  |  |  |
| N-MID, ng/mL | 12.2 (9.6-15.8) | 13.9 (12.4-21.1) | 11.4 (9.3-14.0) | 14.6 (10.1-17.3) | 10.2 (9.4-11.5) | 0.001 | 0.004 |
| P1NP, ng/mL | 44.1 (34.6-57.0) | 47.9 (39.9-65.0) | 39.0 (29.2-48.8) | 49.2 (35.8-63.6) | 48.2 (40.4-53.7) | 0.008 | 0.038 |
| 25(OH)D, ng/mL | 17.7 (13.9-21.2) | 20.5 (13.7-23.2) | 19.7 (15.2-22.8) | 15.8 (13.8-19.5) | 13.1 (11.5-17.7) | 0.004 | 0.021 |
| B-CTX, ng/mL | 0.4 (0.3-0.6) | 0.5 (0.4-0.7) | 0.4 (0.2-0.5) | 0.4 (0.3-0.6) | 0.4 (0.3-0.5) | 0.101 | 0.503 |
| PTH, pg/mL | 39.9 (30.2-49.5) | 33.3 (26.0-48.2) | 38.5 (31.8-46.0) | 44.6 (36.0-57.3) | 43.9 (35.7-56.1) | 0.039 | 0.195 |
| **Bone Mineral Density** |  |  |  |  |  |  |  |
| L1-4 T-score | -0.70 (-1.63-0.20) | -1.20 (-2.30-0.55) | -0.60 (-1.25-0.30) | -0.80 (-1.85-0.05) | -0.40 (-0.78-0.15) | 0.150 | 1.000 |
| L1-4 Z-score | -0.10 (-1.10-0.70) | -0.40 (-1.45-0.85) | -0.50 (-1.10-0.40) | 0.15 (-0.72-0.95) | 0.05 (-0.57-1.62) | 0.173 | 1.000 |
| L Neck T-score | -0.70 (-1.20-0.00) | -1.10 (-1.75--0.55) | -0.65 (-1.00-0.00) | -0.80 (-1.50-0.30) | 0.10 (-0.47-0.57) | 0.000 | 0.001 |
| L Neck Z-score | 0.10 (-0.50-0.70) | -0.30 (-0.80-0.40) | 0.20 (-0.50-0.60) | 0.10 (-0.40-0.90) | 0.65 (0.32-1.25) | 0.001 | 0.009 |
| L Total Hip T-score | -0.35 (-0.80-0.30) | -0.80 (-1.15--0.35) | -0.35 (-0.72-0.20) | -0.30 (-0.95-0.40) | 0.50 (-0.10-0.97) | 0.000 | 0.000 |
| L Total Hip Z-score | 0.10 (-0.50-0.80) | -0.30 (-0.75-0.30) | -0.05 (-0.42-0.50) | 0.30 (-0.42-1.00) | 0.95 (0.02-1.48) | 0.000 | 0.001 |
| R Neck T-score | -0.75 (-1.30-0.18) | -1.20 (-1.72--0.80) | -0.55 (-1.02-0.25) | -0.30 (-1.35-0.25) | 0.00 (-0.40-0.50) | 0.000 | 0.003 |
| R Neck Z-score | 0.20 (-0.40-0.85) | -0.25 (-0.80-0.10) | 0.20 (-0.42-0.85) | 0.20 (-0.40-1.33) | 0.60 (0.40-1.05) | 0.003 | 0.031 |
| R Total Hip T-score | -0.30 (-0.97-0.30) | -0.80 (-1.12--0.27) | -0.40 (-0.90-0.20) | -0.20 (-0.95-0.30) | 0.50 (0.15-0.60) | 0.000 | 0.001 |
| R Total Hip Z-score | 0.10 (-0.50-0.70) | -0.35 (-0.72-0.30) | -0.20 (-0.60-0.60) | 0.25 (-0.35-0.83) | 0.70 (0.65-1.15) | 0.000 | 0.004 |
| Total T-score | -0.90 (-1.60--0.25) | -1.30 (-1.87--0.60) | -0.85 (-1.58--0.10) | -1.10 (-2.10--0.25) | -0.60 (-1.05--0.30) | 0.049 | 0.591 |
| Total Z-score | -0.65 (-1.30-0.00) | -0.90 (-1.30--0.20) | -0.70 (-1.37-0.08) | -0.50 (-1.30-0.00) | -0.50 (-0.80-0.00) | 0.431 | 1.000 |
| Data are median (Q1-Q3) for continuous variables and n (%) for categorical variables. P-values from Kruskal-Wallis rank sum test; adjusted P by Bonferroni correction (P_adj = min[P_raw x N_tests, 1]) within each variable group. - indicates that inferential statistics are not reported for cluster-defining body composition variables, as formal testing would constitute circular inference. Cluster sample sizes: C1=42, C2=71, C3=49, C4=23. | | | | | | | |
