# Supplementary material for: Body Composition Phenotypes and Their Metabolic Associations in Treatment‐Naïve Patients With Newly Diagnosed Type 2 Diabetes: A DXA‐Based Cluster Analysis
Source: Endocrinol Diabetes Metab. 2026 Oct 19;9(6):e70366. doi: 10.1002/edm2.70366 (PMC13680683; doi:10.1002/edm2.70366)
Supplement: Supplementary file 11 [file EDM2-9-e70366-s012.docx]

# Supplemental Table S2. Sex-stratified Kruskal-Wallis tests and sex-by-cluster interaction analyses for key metabolic outcomes.

| **Variable** | **Male Raw P** | **Male Adj P** | **Female Raw P** | **Female Adj P** | **Interaction P** |
| --- | --- | --- | --- | --- | --- |
| ALT(U/L) | 0.0012 | 0.0141 | 0.0612 | 0.7349 | 0.2657 |
| AST(U/L) | 0.0115 | 0.1381 | 0.2726 | 1.0000 | 0.6301 |
| γ-GGT(U/L) | 0.1155 | 1.0000 | 0.0243 | 0.2919 | 0.8573 |
| UA(μmol/L) | 0.0050 | 0.0604 | 0.0035 | 0.0415 | 0.0787 |
| BUN(mmol/L) | 0.0024 | 0.0291 | 0.3109 | 1.0000 | 0.1351 |
| Cr(μmol/L) | 0.9683 | 1.0000 | 0.1448 | 1.0000 | 0.1441 |
| L Total Hip T-score | 0.0005 | 0.0054 | 0.0013 | 0.0159 | 0.0033 |
| L Total Hip Z-score | 0.0163 | 0.1955 | 0.0012 | 0.0145 | 0.0143 |
| HDL-C(mmol/L) | 0.0110 | 0.1322 | 0.0180 | 0.2160 | 0.0952 |
| TG(mmol/L) | 0.0024 | 0.0288 | 0.3364 | 1.0000 | 0.6583 |
| HOMA-IR | 0.0166 | 0.1995 | 0.0033 | 0.0394 | 0.9182 |
| HOMA-β | 0.1387 | 1.0000 | 0.6464 | 1.0000 | 0.6758 |
| Males: Clusters 1, 2, 4 (Cluster 3 excluded, n=1 male). Females: Clusters 1, 3, 4 (Cluster 2 excluded, n=1 female). Adjusted P by Bonferroni (N = 12 tests). Interaction P from linear model ANOVA. | | | | | |
