# Supplementary material for: Body Composition Phenotypes and Their Metabolic Associations in Treatment‐Naïve Patients With Newly Diagnosed Type 2 Diabetes: A DXA‐Based Cluster Analysis
Source: Endocrinol Diabetes Metab. 2026 Oct 19;9(6):e70366. doi: 10.1002/edm2.70366 (PMC13680683; doi:10.1002/edm2.70366)
Supplement: Supplementary file 12 [file EDM2-9-e70366-s013.docx]

# Supplemental Table S7. Sensitivity analysis: complete-case vs multiple imputation logistic regression for complication outcomes.

| **Outcome** | **Term** | **CC OR** | **CC 95% CI** | **CC P** | **MI OR** | **MI 95% CI** | **MI P** |
| --- | --- | --- | --- | --- | --- | --- | --- |
| Osteoporosis | === CC:N=156, MI:N=186 === |  |  |  |  |  |  |
|  | Intercept | 0.04 | 0.00-0.36 | 0.0041 | 0.04 | 0.00-0.30 | 0.0018 |
|  | Cluster 2 vs 1 | 0.97 | 0.33-2.86 | 0.9629 | 1.23 | 0.42-3.61 | 0.7076 |
|  | Cluster 3 vs 1 | 0.62 | 0.15-2.59 | 0.5147 | 0.65 | 0.18-2.34 | 0.5068 |
|  | Cluster 4 vs 1 | 0.15 | 0.03-0.90 | 0.0379 | 0.16 | 0.03-0.92 | 0.0395 |
|  | Age (per year) | 1.07 | 1.03-1.10 | <0.001 | 1.07 | 1.04-1.10 | <0.001 |
|  | Male sex | 0.16 | 0.03-0.76 | 0.0217 | 0.14 | 0.03-0.66 | 0.0131 |
|  | Current smoking | 5.36 | 1.68-17.16 | 0.0046 | 5.30 | 1.68-16.76 | 0.0046 |
|  | Current drinking | 0.59 | 0.21-1.67 | 0.3249 | 0.62 | 0.23-1.69 | 0.3471 |
|  |  |  |  |  |  |  |  |
| NAFLD | === CC:N=142, MI:N=186 === |  |  |  |  |  |  |
|  | Intercept | 0.39 | 0.05-2.92 | 0.3581 | 0.37 | 0.06-2.34 | 0.2895 |
|  | Cluster 2 vs 1 | 11.68 | 3.91-34.93 | <0.001 | 9.69 | 3.34-28.09 | <0.001 |
|  | Cluster 3 vs 1 | 2.19 | 0.53-9.02 | 0.2760 | 2.44 | 0.74-8.02 | 0.1418 |
|  | Cluster 4 vs 1 | 9.52 | 2.33-38.92 | 0.0017 | 8.55 | 2.18-33.51 | 0.0021 |
|  | Age (per year) | 1.01 | 0.98-1.03 | 0.6311 | 1.01 | 0.98-1.03 | 0.6529 |
|  | Male sex | 0.69 | 0.16-2.94 | 0.6135 | 1.02 | 0.28-3.67 | 0.9778 |
|  | Current smoking | 0.67 | 0.24-1.82 | 0.4277 | 0.62 | 0.23-1.67 | 0.3433 |
|  | Current drinking | 1.70 | 0.62-4.68 | 0.3047 | 1.57 | 0.54-4.52 | 0.4085 |
|  |  |  |  |  |  |  |  |
| Hyperuricemia | === CC:N=156, MI:N=186 === |  |  |  |  |  |  |
|  | Intercept | 1.26 | 0.14-11.16 | 0.8343 | 0.66 | 0.08-5.51 | 0.7012 |
|  | Cluster 2 vs 1 | 3.65 | 0.83-16.13 | 0.0877 | 3.99 | 0.91-17.40 | 0.0659 |
|  | Cluster 3 vs 1 | 1.39 | 0.27-7.22 | 0.6969 | 1.40 | 0.29-6.71 | 0.6734 |
|  | Cluster 4 vs 1 | 4.29 | 0.93-19.78 | 0.0621 | 6.36 | 1.42-28.57 | 0.0158 |
|  | Age (per year) | 0.97 | 0.94-1.00 | 0.0227 | 0.97 | 0.94-1.00 | 0.0489 |
|  | Male sex | 0.34 | 0.07-1.52 | 0.1569 | 0.37 | 0.09-1.57 | 0.1789 |
|  | Current smoking | 0.81 | 0.29-2.27 | 0.6941 | 0.86 | 0.31-2.40 | 0.7715 |
|  | Current drinking | 1.06 | 0.38-2.96 | 0.9138 | 1.14 | 0.41-3.18 | 0.8020 |
|  |  |  |  |  |  |  |  |
| LEAD | === CC:N=117, MI:N=186 === |  |  |  |  |  |  |
|  | Intercept | 0.00 | 0.00-0.01 | <0.001 | 0.00 | 0.00-0.04 | <0.001 |
|  | Cluster 2 vs 1 | 0.51 | 0.10-2.46 | 0.3980 | 0.58 | 0.14-2.41 | 0.4517 |
|  | Cluster 3 vs 1 | 1.03 | 0.12-9.08 | 0.9753 | 1.00 | 0.15-6.61 | 0.9973 |
|  | Cluster 4 vs 1 | 0.53 | 0.09-2.96 | 0.4683 | 0.55 | 0.11-2.68 | 0.4629 |
|  | Age (per year) | 1.18 | 1.11-1.25 | <0.001 | 1.14 | 1.08-1.21 | <0.001 |
|  | Male sex | 8.43 | 0.99-71.75 | 0.0509 | 3.71 | 0.73-18.87 | 0.1154 |
|  | Current smoking | 1.07 | 0.30-3.85 | 0.9116 | 1.38 | 0.40-4.68 | 0.6105 |
|  | Current drinking | 0.35 | 0.09-1.35 | 0.1278 | 0.46 | 0.13-1.67 | 0.2407 |
|  |  |  |  |  |  |  |  |
| DKD | === CC:N=156, MI:N=186 === |  |  |  |  |  |  |
|  | Intercept | 42.69 | 2.94-620.99 | 0.0060 | 31.93 | 3.47-293.69 | 0.0022 |
|  | Cluster 2 vs 1 | 0.50 | 0.12-2.04 | 0.3336 | 0.50 | 0.16-1.63 | 0.2539 |
|  | Cluster 3 vs 1 | 1.33 | 0.18-9.66 | 0.7764 | 1.06 | 0.25-4.50 | 0.9398 |
|  | Cluster 4 vs 1 | 1.06 | 0.22-5.12 | 0.9449 | 0.63 | 0.16-2.54 | 0.5192 |
|  | Age (per year) | 0.89 | 0.86-0.93 | <0.001 | 0.91 | 0.88-0.95 | <0.001 |
|  | Male sex | 0.77 | 0.13-4.61 | 0.7731 | 0.60 | 0.14-2.58 | 0.4894 |
|  | Current smoking | 1.43 | 0.45-4.52 | 0.5452 | 1.61 | 0.50-5.12 | 0.4238 |
|  | Current drinking | 1.25 | 0.39-3.93 | 0.7076 | 1.13 | 0.38-3.38 | 0.8216 |
|  |  |  |  |  |  |  |  |
| DR | === CC:N=96, MI:N=186 === |  |  |  |  |  |  |
|  | Intercept | 0.03 | 0.00-1.74 | 0.0893 | 0.06 | 0.00-2.62 | 0.1476 |
|  | Cluster 2 vs 1 | 1.22 | 0.18-8.34 | 0.8366 | 1.45 | 0.25-8.59 | 0.6813 |
|  | Cluster 3 vs 1 | 1.04 | 0.04-24.86 | 0.9790 | 1.24 | 0.10-14.74 | 0.8642 |
|  | Cluster 4 vs 1 | 1.63 | 0.11-24.28 | 0.7228 | 3.89 | 0.47-32.22 | 0.2098 |
|  | Age (per year) | 1.02 | 0.96-1.08 | 0.4839 | 1.01 | 0.95-1.06 | 0.8176 |
|  | Male sex | 1.76 | 0.12-26.63 | 0.6846 | 1.59 | 0.17-14.54 | 0.6810 |
|  | Current smoking | 0.65 | 0.11-3.93 | 0.6373 | 0.50 | 0.10-2.37 | 0.3828 |
|  | Current drinking | 0.33 | 0.03-3.37 | 0.3497 | 0.49 | 0.05-5.07 | 0.5484 |
|  |  |  |  |  |  |  |  |
| All models: outcome ~ cluster + age + sex + smoking + drinking. Reference: Cluster 1, Female sex, Non-smoker, Non-drinker. MI: 20 imputed datasets (MICE), pooled by Rubin rules. CC: Complete-case analysis. | | | | | | | |
